# METTL3-dependent m^6^A modification programs T follicular helper cell differentiation

**DOI:** 10.1038/s41467-021-21594-6

**Published:** 2021-02-26

**Authors:** Yingpeng Yao, Ying Yang, Wenhui Guo, Lifan Xu, Menghao You, Yi-Chang Zhang, Zhen Sun, Xiao Cui, Guotao Yu, Zhihong Qi, Jingjing Liu, Fang Wang, Juanjuan Liu, Tianyan Zhao, Lilin Ye, Yun-Gui Yang, Shuyang Yu

**Affiliations:** 1 State Key Laboratory of Agrobiotechnology, College of Biological Sciences, China Agricultural University, Beijing, China; 2 CAS Key Laboratory of Genomic and Precision Medicine, Collaborative Innovation Center of Genetics and Development, CAS Center for Excellence in Molecular Cell Science, College of Future Technology, Beijing Institute of Genomics, Chinese Academy of Sciences, Beijing, China; 3 University of Chinese Academy of Sciences, Beijing, China; 4 Institute of Immunology, Third Military Medical University, Chongqing, China

**Keywords:** Lymphocyte differentiation, Follicular T-helper cells

## Abstract

T follicular helper (T_FH_) cells are specialized effector CD4^+^ T cells critical to humoral immunity. Whether post-transcriptional regulation has a function in T_FH_ cells is unknown. Here, we show conditional deletion of METTL3 (a methyltransferase catalyzing mRNA *N*^6^-methyladenosine (m^6^A) modification) in CD4^+^ T cells impairs T_FH_ differentiation and germinal center responses in a cell-intrinsic manner in mice. METTL3 is necessary for expression of important T_FH_ signature genes, including *Tcf7*, *Bcl6*, *Icos* and *Cxcr5* and these effects depend on intact methyltransferase activity. m^6^A-miCLIP-seq shows the 3′ UTR of *Tcf7* mRNA is subjected to METTL3-dependent m^6^A modification. Loss of METTL3 or mutation of the *Tcf7* 3′ UTR m^6^A site results in accelerated decay of *Tcf7* transcripts. Importantly, ectopic expression of TCF-1 (encoded by *Tcf7*) rectifies T_FH_ defects owing to METTL3 deficiency. Our findings indicate that METTL3 stabilizes *Tcf7* transcripts via m^6^A modification to ensure activation of a T_FH_ transcriptional program, indicating a pivotal function of post-transcriptional regulation in promoting T_FH_ cell differentiation.

## Abstract (web summary)

T follicular helper (T_FH_) cells are specialized effector CD4^+^ T cells that are critical in humoral immunity, but the function of post-transcriptional regulation is not clearly defined. Here, the authors demonstrate that RNA methylation is important for T_FH_ effector differentiation and subsequent antibody formation through stabilization of *Tcf7* transcripts.

## Introduction

The production of high-affinity antibodies and generation of memory cells are critical for establishing protective immunity^1,2^. During acute viral infection, T follicular helper (T_FH_) cells provide help to cognate antigen-presenting B cells to facilitate the formation of germinal centers (GCs) and the development of long-lived plasma cells and memory B cells^3^. After priming by dendritic cells (DCs) in the T-cell zone, CD4^+^ T cells upregulate the expression of chemokine receptor CXCR5, the costimulatory receptor ICOS and the transcriptional repressor B-cell lymphoma 6 (Bcl-6), which together coordinate early fate commitment of T_FH_ cells and their migration to the follicles. After interacting with cognate antigen-presenting B cells, these activated CD4^+^ T cells mature into T_FH_ cells and further differentiate into GC T_FH_ cells, providing help to GC B cells for humoral immunity^4,5^.

T_FH_ differentiation is a complicated biological process^6^, which is tightly controlled by multiple transcription factors. As a master regulator of T_FH_ differentiation, Bcl-6 represses the T_H_1, T_H_2, and T_H_17 lineage-specific transcription factors to promote the differentiation of activated CD4^+^ T cells into T_FH_ cells^7–9^. On the contrary, Blimp1 (encoded by *Prdm1*) negatively modulates Bcl-6 transcription by binding to the *Bcl6* promoter and acting as a repressor, thus preventing T_FH_ differentiation and promoting the formation of non-T_FH_ effector helper T cells^9,10^. Accumulative studies have demonstrated that TCF-1 (encoded by *Tcf7*) plays key roles in T_FH_ differentiation by directly acting upstream of the Bcl-6–Blimp1 axis^11–13^. Although the regulation of these transcription factors has been intensely investigated at the transcriptional level, it remains unknown if post transcriptional mechanisms are involved in the balanced expression of these key factors during T_FH_ differentiation.

*N*^6^-methyladenosine (m^6^A) is the most prevalent modification on eukaryote mRNAs, catalyzed by m^6^A methyltransferases^14^. Methyltransferase like 3 protein (METTL3, encoded by *Mettl3*) is the core catalytic subunit of m^6^A methyltransferases, and METTL14 is an allosteric activator of METTL3^15,16^. The recruitment of m^6^A-binding proteins is actively involved in almost every stage of mRNA metabolism, from processing in the nucleus to translation and decay in the cytoplasm, adding another layer of regulatory mechanisms in gene expression^17^. Early studies showed that m^6^A-tagged transcripts had shorter half-life^18^, and binding of YTHDF2 to m^6^A promoted m^6^A-modified mRNA decay^18,19^. Recently, it was discovered that a group of novel m^6^A-binding proteins, IGF2BPs, stabilized methylated transcripts by guarding them against degradation^20,21^. The distinct roles of YTHDF2 and IGF2BPs suggest that the impact of m^6^A modification on mRNA stability is highly dependent on cell context.

Several studies have uncovered critical roles of m^6^A modification in immune regulation. It has been documented that m^6^A methylation is essential for normal hematopoiesis and leukemia development^22–24^. m^6^A methylation regulates T-cell homeostasis, as well as suppressive functions of Treg cells by targeting *Socs* genes^25,26^. In the context of viral infection, m^6^A represses type I interferon production in an innate antiviral state^27,28^. Despite these profound effects of m^6^A modification on immunoregulation, its role in T_FH_ differentiation has not been determined.

In this study, by conditional targeting the *Mettl3* gene in T cells, we demonstrate that METTL3-mediated m^6^A modification is critical for T_FH_ cell differentiation. Mechanistically, METTL3 deficiency impairs the stability of m^6^A-modified *Tcf7* mRNA, resulting in compromised activation of T_FH_ transcriptional program.

## Results

### METTL3 controls T_FH_ differentiation and GC reactions

**Fig. 1:**
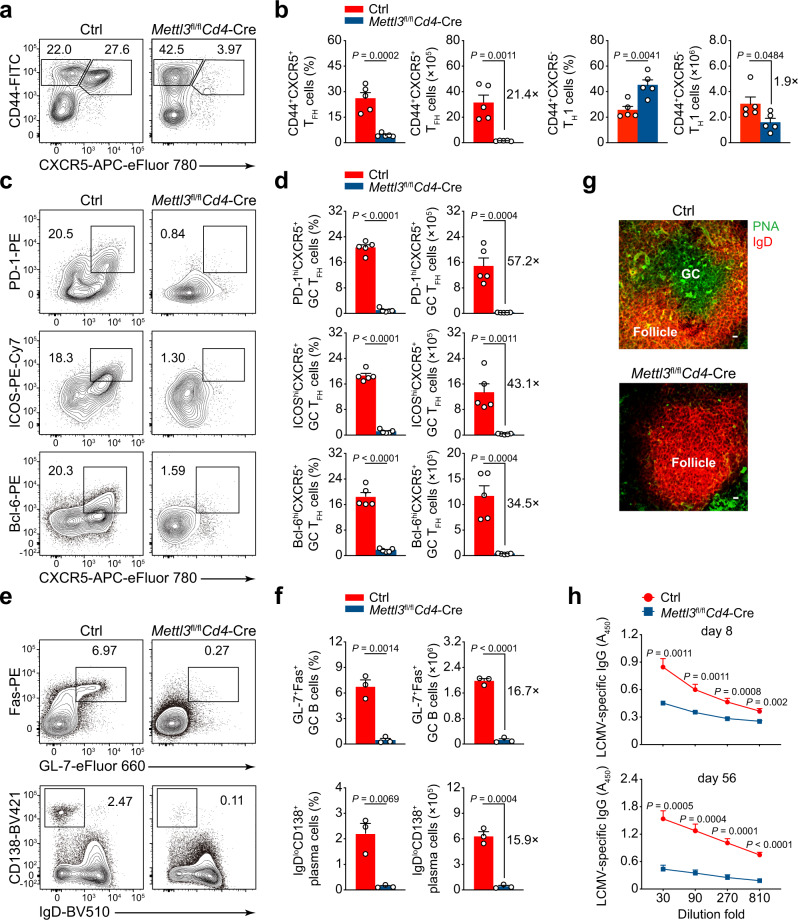
Conditional ablation of METTL3 impairs T_FH_ differentiation and GC responses. **a**, **b** Flow cytometry analysis of CD44^+^CXCR5^+^ T_FH_ cells and CD44^+^CXCR5^–^ T_H_1 cells, gated on splenic CD4^+^ T cells from Ctrl and *Mettl3*^fl/fl^*Cd4*-Cre mice on day 8 post infection (8 *dpi*). Summary of the frequency and cell numbers of indicated cell subsets are shown in **b** (*n* = 5 per group). **c**, **d** Flow cytometry analysis of PD-1^hi^CXCR5^+^ GC T_FH_ cells (top panel), ICOS^hi^CXCR5^+^ GC T_FH_ cells (middle panel), and Bcl-6^hi^CXCR5^+^ GC T_FH_ cells (bottom panel), gated on splenic CD44^hi^CD62L^lo^CD4^+^ T cells from Ctrl and *Mettl3*^fl/fl^*Cd4*-Cre mice on 8 *dpi*. Summary of the frequency and cell numbers of indicated cell subsets are shown in **d** (*n* = 5 per group). **e**, **f** Flow cytometry analysis of splenic GL-7^+^Fas^+^ GC B cells (top panel) and IgD^lo^CD138^+^ plasma cells (bottom panel) on 8 *dpi*. Summary of the frequency and cell numbers of GC B cells and plasma cells are shown in **f** (*n* = 3 per group). **g** Immunofluorescent staining of spleens from Ctrl and *Mettl3*^fl/fl^*Cd4*-Cre on 8 *dpi*. Green: PNA; Red: IgD; scale bar: 10 μm. **h** Analysis of LCMV-specific IgG concentration in serum on 8 *dpi* (top) and on 56 *dpi* (bottom) by ELISA (day 8: *n* = 12 for Ctrl group, *n* = 10 for *Mettl3*^fl/fl^*Cd4*-Cre group; day 56: *n* = 5 per group). Data are representative of at least three independent experiments. Error bars indicate standard error of the mean. *P* value was calculated by unpaired two-tailed Student’s *t* test.

To investigate the role of METTL3 in T_FH_ differentiation, *Mettl3*-floxed mice were crossed with *Cd4*-Cre mice to generate conditional deletion of METTL3 in T cells (*Mettl3*^fl/fl^*Cd4*-Cre mice), and the deletion efficiency of METTL3 in splenic CD4^+^ T cells was validated by quantitative RT-PCR (Supplementary Fig. 1a). We then infected *Mettl3*^fl/fl^*Cd4*-Cre mice and their wild-type control littermates (Ctrl) with LCMV-Armstrong strain. The frequency and numbers of CD44^+^CXCR5^+^ T_FH_ cells were significantly diminished in *Mettl3*^fl/fl^*Cd4*-Cre mice compared with those in their control littermates on day 8 post viral infection (Fig. 1a, b). In contrast, METTL3-deficient CD4^+^ T cells were dramatically skewed toward the CD44^+^CXCR5^−^ T_H_1 proportion, albeit the numbers of T_H_1 cells were also decreased with the ablation of METTL3 (Fig. 1a, b). It is worth mentioning that ablation of METTL3 resulted in more severe defects in T_FH_ cells (21.4-fold change) than T_H_1 cells (1.9-fold change) upon acute viral infection (Fig. 1b). Given T-bet is expressed at a relatively high level on T_H_1 lineage and directs its commitment^29^, we also analyzed T-bet expression on both T_H_1 and T_FH_ cells. We found T_H_1 cells expressed a much higher level of T-bet than T_FH_ cells, and both T_FH_ and T_H_1 cells downregulated T-bet expression in the absence of METTL3 (Supplementary Fig. 1b). Moreover, *Mettl3*^fl/fl^*Cd4*-Cre mice exhibited remarkably lower percentages and absolute cell numbers of GC T_FH_ cells (identified as PD-1^hi^CXCR5^+^, ICOS^hi^CXCR5^+^, or Bcl-6^hi^CXCR5^+^) than those of their control littermates (Fig. 1c, d). Accordingly, the expression levels of CXCR5, PD-1, ICOS, and Bcl-6 were dramatically lower on METTL3-deficient T_FH_ cells than on wild-type cells (Supplementary Fig. 1c, d). These results indicated that METTL3 deficiency severely impairs T_FH_ differentiation.

Given the main function of T_FH_ cells is to provide cognate B-cell help, which is a fundamental aspect of humoral immunity and generation of immunological memory^1^. We next examined whether METTL3 deficiency in CD4^+^ T cells affects GC formation during viral infection. A robust reduction in the proportions and numbers of GL-7^+^Fas^+^ GC B cells (Fig. 1e, f) and PNA^+^Fas^+^ GC B cells (Supplementary Fig. 1e, f) were observed in *Mettl3*^fl/fl^*Cd4*-Cre mice, compared with those in their wild-type counterparts. Furthermore, the frequency and cell numbers of IgD^lo^CD138^+^ plasma cells were also much lower in *Mettl3*^fl/fl^*Cd4*-Cre mice than those in wild-type mice (Fig. 1e, f). The immunohistochemical analysis further confirmed substantially reduced PNA^+^ GCs within B-cell follicles in spleens from *Mettl3*^fl/fl^*Cd4*-Cre mice (Fig. 1g). To assess the consequences of defective T_FH_ and GC responses in *Mettl3*^fl/fl^*Cd4*-Cre mice, we measured the LCMV-specific serum concentration of immunoglobulin G (IgG). The LCMV-specific IgG concentration was significantly lower in *Mettl3*^fl/fl^*Cd4*-Cre mice than that in their wild-type control littermates on day 8 and day 56 post viral infection (Fig. 1h). Collectively, these results suggested that METTL3 is required for T_FH_ differentiation and GC responses.

To further validate these findings, we immunized *Mettl3*^fl/fl^*Cd4*-Cre mice and their wild-type control littermates by intraperitoneal administration of keyhole limpet hemocyanin (KLH) emulsified in Complete Freund’s Adjuvant (CFA). On day 8 post immunization, *Mettl3*^fl/fl^*Cd4*-Cre mice exhibited impaired development of T_FH_ cells and GC T_FH_ cells, but not T_H_1 cells (Supplementary Fig. 2a, b). Meanwhile, the percentages and cell numbers of GC B cells and plasma cells (Supplementary Fig. 2c, d) were also impaired in *Mettl3*^fl/fl^*Cd4*-Cre mice compared with their wild-type counterparts. These data jointly indicated METTL3 promotes T_FH_ differentiation upon different antigen stimulation.

Meanwhile, the differentiation of other T helper lineages was also examined by using KLH immunization model. We found that both GATA3^+^ and IL-4-producing cells, as well as Foxp3^+^ cells were not altered in *Mettl3*^fl/fl^*Cd4*-Cre mice (Supplementary Fig. 2e–h), indicating T_H_2 and Treg cell differentiation are not affected in the absence of METTL3 upon KLH immunization. Interestingly, both RORγt^+^ and IL-17a-producing cells were significantly decreased in METTL3-deficient mice (Supplementary Fig. 2e–h), revealing METTL3 is essential for T_H_17 cell differentiation in vivo upon protein immunization.

### METTL3 intrinsically regulates T_FH_ differentiation

We next focused on whether METTL3 regulates T_FH_ differentiation in a cell-intrinsic manner using both bone marrow chimeric and adoptive transfer mice models. We first generated bone marrow chimeric mice by reconstituting lethally irradiated wild-type recipient mice (CD45.1^+^) with a mixture donor of bone marrow cells from *Mettl3*^fl/fl^*Cd4*-Cre mice (CD45.2^+^) and wild-type (CD45.1^+^) competitor mice (Supplementary Fig. 3a). On day 8 post LCMV-Armstrong infection, *Mettl3*^fl/fl^*Cd4*-Cre mice-derived CD4^+^ T cells had much lower cell numbers of CD44^+^CXCR5^+^ T_FH_ cells than those of competitor bone marrow-derived CD4^+^ T cells (Supplementary Fig. 3b, c). Correspondingly, CD4^+^ T cells originated in *Mettl3*^fl/fl^*Cd4*-Cre mice also exhibited a reduction of PD-1^hi^CXCR5^+^ GC T_FH_ cells (Supplementary Fig. 3b, c). The expression levels of CXCR5, PD-1, ICOS, and Bcl-6 were significantly lower on METTL3-deficient T_FH_ cells than those on competitor cells (Supplementary Fig. 3d).

**Fig. 2:**
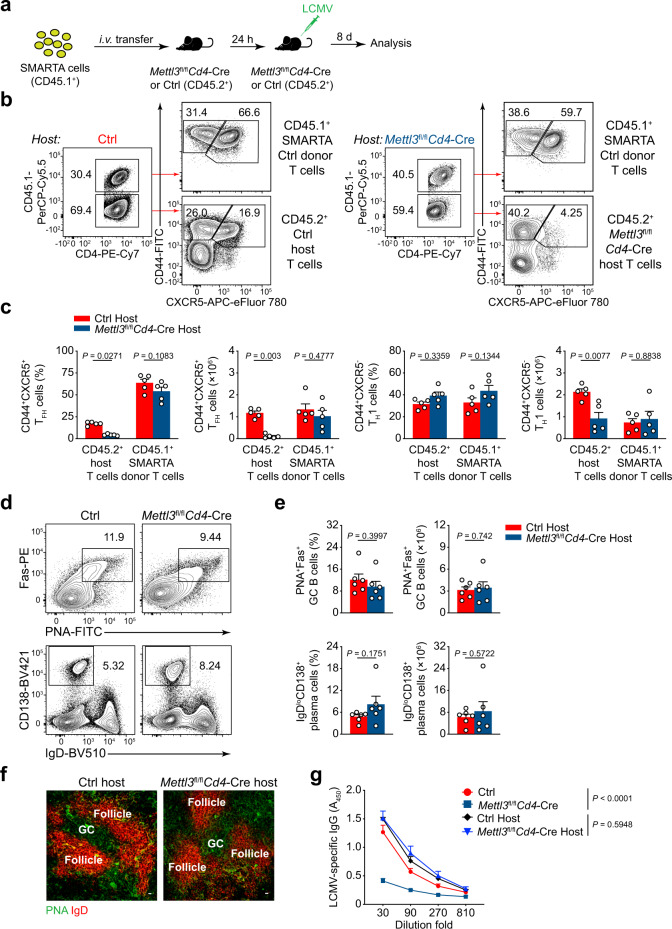
METTL3 intrinsically controls T_FH_ differentiation and GC responses. **a** Scheme of adoptive transfer model. 5 × 10^6^ CD45.1^+^ SMARTA cells were adoptively transferred into CD45.2^+^ *Mettl3*^fl/fl^*Cd4*-Cre mice or Ctrl host mice, followed by LCMV-Armstrong infection within 24 h. **b**, **c** Flow cytometry analysis of CD45.1^+^ SMARTA or CD45.2^+^ host T cell-derived CD44^+^CXCR5^+^ T_FH_ and CD44^+^CXCR5^–^ T_H_1 cells, gated on splenic CD4^+^ T cells from host mice on day 8 post viral infection. Frequency and numbers of CD44^+^CXCR5^+^ T_FH_ cells and CD44^+^CXCR5^–^ T_H_1 cells are shown in **c** (*n* = 5 per group). **d**, **e** Flow cytometry analysis of splenic PNA^+^Fas^+^ GC B cells (top panel) and IgD^lo^CD138^+^ plasma cells (bottom panel) from host mice on 8 *dpi*. Summary of the frequency and cell numbers of GC B cells and plasma cells are shown in **e** (*n* = 6 per group). **f** Immunofluorescent staining of spleens from Ctrl or *Mettl3*^fl/fl^*Cd4*-Cre host mice on 8 *dpi*. Green: PNA; Red: IgD; scale bar: 10 μm. **g** Analysis of LCMV-specific IgG concentration in serum on 8 *dpi* by ELISA (*n* = 5 for Ctrl group, *n* = 5 for *Mettl3*^fl/fl^*Cd4*-Cre group, *n* = 6 for Ctrl host group, *n* = 6 for *Mettl3*^fl/fl^*Cd4*-Cre host group). Data are representative of at least three independent experiments. Error bars indicate standard error of the mean. *P* value was calculated by unpaired two-tailed Student’s *t* test (**e**) or two-way ANOVA coupled with multiple comparisons (**c**, **g**).

To further rule out the effect of external factors on T_FH_ differentiation, we adoptively transferred CD45.1^+^ SMARTA CD4^+^ T cells into CD45.2^+^ *Mettl3*^fl/fl^*Cd4*-Cre or their wild-type control littermates (Fig. 2a). On day 8 post viral infection, we observed the percentages and numbers of CD44^+^CXCR5^+^ T_FH_ cells derived from CD45.1^+^ SMARTA CD4^+^ T cells were similar in the *Mettl3*^fl/fl^*Cd4*-Cre mice and their wild-type counterparts (Fig. 2b, c), indicating the microenvironment in the *Mettl3*^fl/fl^*Cd4*-Cre mice did not impair T_FH_ differentiation. In contrast, METTL3-deficient CD4^+^ T cells displayed compromised T_FH_ differentiation compared with that of congenic wild-type CD4^+^ T cells (Fig. 2b, c). Correspondingly, METTL3-deficient CD4^+^ T cells also exhibited defects in their ability to differentiate into CD44^+^CXCR5^−^ T_H_1 cells (Fig. 2b, c). Besides, transferred CD45.1^+^ SMARTA cells also profoundly promoted the GC B and plasma cells differentiation (Fig. 2d, e), GC formation (Fig. 2f), and excessive production of LCMV-specific IgG antibody (Fig. 2g) in *Mettl3*^fl/fl^*Cd4*-Cre recipients. Together, our data demonstrated METTL3 is intrinsically required for T_FH_ cell differentiation and functions as a key role in GCs formation.

### METTL3 is essential for the early initiation of T_FH_ cells

**Fig. 3:**
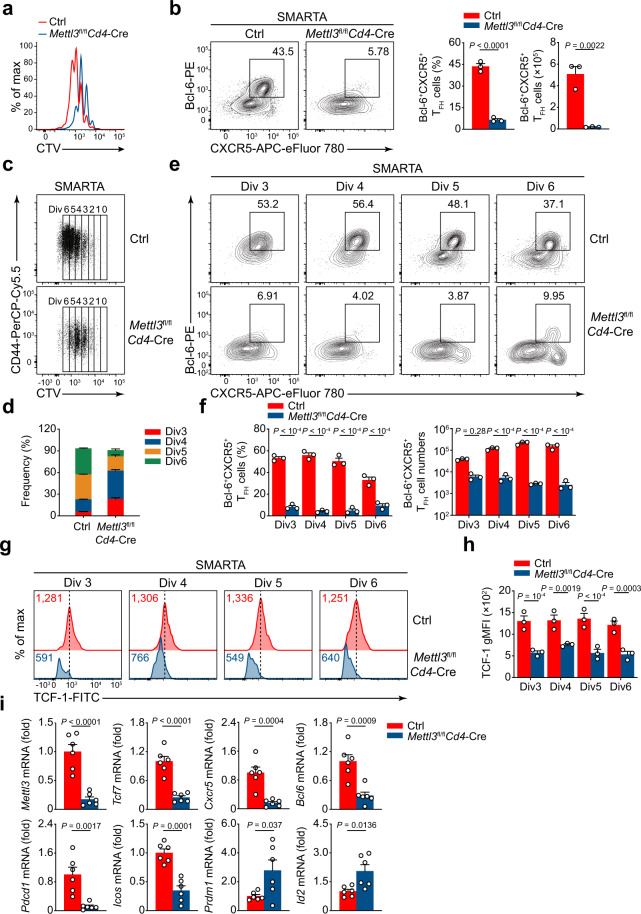
METTL3 is indispensable for the initiation of T_FH_ development. **a** Flow cytometry analysis of cells from the wild-type recipient mice (CD45.1^+^) given adoptive transfer of naive CTV-labeled Ctrl or *Mettl3*^fl/fl^*Cd4*-Cre SMARTA cells, followed by LCMV-Armstrong infection and analysis 3 days later as CTV dilution by the transferred cells. **b** Flow cytometry analysis of Bcl-6^+^CXCR5^+^ T_FH_ cells gated on SMARTA CD4^+^ T cells from recipient mice as in **a**. Summary of the frequency and cell numbers of T_FH_ cells are shown on the right (*n* = 3 per group). **c**, **d** Flow cytometry analysis of different cell divisions gated on SMARTA CD4^+^ T cells from recipient mice as in **a**. The frequency of third to sixth divisions is summarized in **d** (*n* = 3 per group). **e**, **f** Contour plots display Bcl-6^+^CXCR5^+^ T_FH_ cells in indicated divisions, among SMARTA cells from recipient mice as in **a**. Summary of the frequency and cell numbers of T_FH_ cells in indicated divisions are shown in **f** (*n* = 3 per group). **g**, **h** Flow cytometry analysis of the expression of TCF-1 in CXCR5^+^ cells in indicated divisions gated on SMARTA CD4^+^ T cells from recipient mice as in **a**. Quantification of geometric mean fluorescence intensity (gMFI) of TCF-1 in indicated divisions is shown in **h** (*n* = 3 per group). **i** Quantitative RT-PCR analysis of mRNA abundance of T_FH_ cell-related genes in CD25^–^CXCR5^+^ T_FH_ cells from recipient mice as in **a**, relative expression was normalized to Ctrl cells (*n* = 6 per group). Data are representative of two independent experiments. Error bars indicate standard error of the mean. *P* value was calculated by unpaired two-tailed Student’s *t* test (**b**, **i**) or two-way ANOVA coupled with multiple comparisons (**f**, **h**).

During acute viral infection, effector CD4^+^ T cells’ commitment to the T_FH_ lineage or T_H_1 lineage emerges before the initiation of GCs^12^. To investigate whether METTL3 is required for early T_FH_ specification in vivo, naive Ctrl or *Mettl3*^fl/fl^*Cd4*-Cre SMARTA CD4^+^ T cells were labeled with cell-trace violet (CTV) and adoptively transferred into congenic recipient mice, followed by LCMV-Armstrong infection (Supplementary Fig. 4a). On day 3 post viral infection, SMARTA CD4^+^ T cells of both genotypes showed similar upregulation of T-cell activation markers CD69 and CD44, and downregulation of CD62L (Supplementary Fig. 4b, c). In vitro cell culture analysis also revealed that *Mettl3*^fl/fl^*Cd4*-Cre SMARTA CD4^+^ T cells displayed no obvious defects in T-cell activation (Supplementary Fig. 4d). Ctrl SMARTA CD4^+^ T cells exhibited vigorously proliferation post viral infection, whereas METTL3-deficient cells displayed a delayed proliferation (Fig. 3a). Lineage commitment to T_FH_ lineage has occurred on day 3 post viral infection, as detected by Bcl-6^+^CXCR5^+^ cells^30^. Consistently, wild-type SMARTA cells developed robust numbers of Bcl-6^+^CXCR5^+^ T_FH_ cells, whereas METTL3-deficient cells exhibited much lower percentages and cell numbers of Bcl-6^+^CXCR5^+^ T_FH_ cells (Fig. 3b). Tracking the cell division showed *Mettl3*^fl/fl^*Cd4*-Cre SMARTA cells were predominantly in third and fourth divisions, while Ctrl SMARTA cells had advanced to fifth and sixth divisions (Fig. 3c, d), reflecting the impaired proliferation of activated CD4^+^ T cells in the absence of METTL3. Consistently, *Mettl3*^fl/fl^*Cd4*-Cre SMARTA cells exhibited a reduction of proportions and cell numbers of Bcl-6^+^CXCR5^+^ T_FH_ cells in indicated cell divisions compared with that of Ctrl SMARTA cells (Fig. 3e, f). Interestingly, as a critical regulator for early T_FH_ differentiation^31^, TCF-1 expression in CXCR5^+^ cells was also dramatically reduced due to ablation of METTL3 (Fig. 3g, h). Moreover, we also observed compromised CD25^−^CXCR5^+^ T_FH_ cells in *Mettl3*^fl/fl^*Cd4*-Cre SMARTA cells by using the combination of CXCR5 and CD25 to identify T_FH_ cells in activated CD4^+^ T cells (Supplementary Fig. 4e, f), and apoptosis of T_FH_ cells was elevated in the absence of METTL3 (Supplementary Fig. 4g, h). We further assessed the expression levels of genes that are associated with T_FH_ cells, including *Tcf7*, *Cxcr5*, *Bcl6*, *Pdcd1*, and *Icos*. The expression levels of these genes were substantially decreased in *Mettl3*^fl/fl^*Cd4*-Cre SMARTA T_FH_ cells compared with those of Ctrl cells (Fig. 3i). Correspondingly, the expressions of *Prdm1* and *Id2*, both well-known for promoting T_H_1 differentiation^9,32^, were much lower in Ctrl T_FH_ cells than that of *Mettl3*^fl/fl^*Cd4*-Cre SMARTA cells (Fig. 3i). These results thus demonstrated that METTL3 is indispensable for early T_FH_ commitment during acute viral infection.

### METTL3 orchestrates the transcriptional profiles of T_FH_ cells

**Fig. 4:**
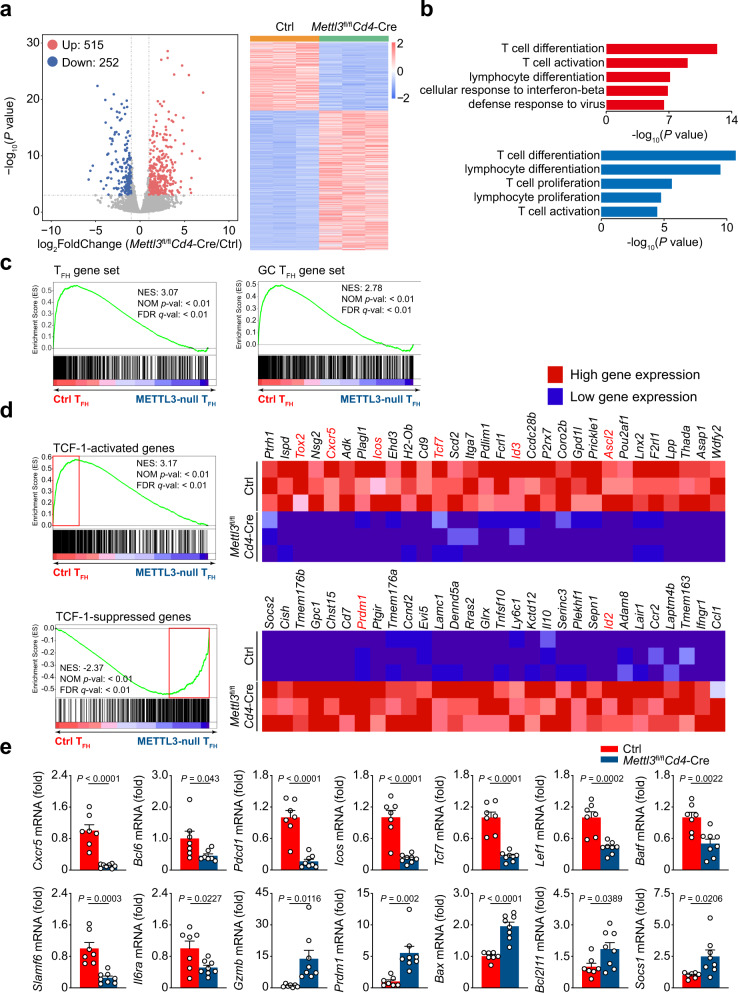
Alternation of transcriptional profiles in METTL3-null T_FH_ cells. **a** Volcano map depicting genes upregulated (red) or downregulated (blue) 2-fold or more in T_FH_ cells on 8 *dpi*. Heatmap of differentially expressed genes is shown on the right. **b** Gene Ontology (GO) terms of the differentially expressed genes in *Mettl3*^fl/fl^*Cd4*-Cre T_FH_ cells compared with Ctrl T_FH_ cells (Upregulated: top panel; Downregulated: bottom panel). **c** Gene set enrichment analysis (GSEA) of T_FH_ and GC T_FH_ gene set in *Mettl3*^fl/fl^*Cd4*-Cre T_FH_ cells relative to expression in Ctrl T_FH_ cells as in **a**. **d** GSEA analysis of “TCF-1-activated genes in T_FH_ cells” and “TCF-1-suppressed genes in T_FH_ cells” (GSE65693) in *Mettl3*^fl/fl^*Cd4*-Cre T_FH_ cells relative to expression in Ctrl T_FH_ cells as in **a**. Heatmaps representation of top 30 ranking genes in the leading edge are shown, respectively. **e** Quantitative RT-PCR analysis of selected interested genes in **a**, relative expression was normalized to that in Ctrl T_FH_ cells (*n* = 7 for Ctrl group, *n* = 8 for *Mettl3*^fl/fl^*Cd4*-Cre group except *Bcl6* gene (*n* = 7 per group)). Data are from one experiment with triplicates (**a**–**d**) or pooled from two experiments (**e**). Error bars indicate standard error of the mean. *P* value was calculated by unpaired two-tailed Student’s *t* test (**e**).

We next investigated how METTL3 deficiency affects the transcriptional profiles of T_FH_ cells. Considering CD4^+^ T cells generally differentiate into T_H_1 cells or T_FH_ cells upon acute viral infection^33^ and METTL3 deficiency affects CXCR5 expression, we hence used CD44 and SLAM, which is expressed at low level on T_FH_ cells^9^, to identify T_H_1 and T_FH_ cells in activated CD4^+^ T cells. CD44^+^SLAM^hi^ T_H_1 cells and CD44^+^SLAM^lo^ T_FH_ cells were sorted from *Mettl3*^fl/fl^*Cd4*-Cre mice and their Ctrl littermates on day 8 post viral infection and subjected to RNA-seq analysis. Compared with those in wild-type cells, 515 upregulated genes and 252 downregulated genes in METTL3-deficient T_FH_ cells (Fig. 4a) and 763 upregulated genes and 332 downregulated genes (Supplementary Fig. 5a) in METTL3-deficient T_H_1 cells were identified, respectively (≥2-fold expression change, adjusted *P* value <0.01). In particular, the upregulated genes in T_FH_ cells were significantly enriched in the T-cell differentiation and defense response to virus; the downregulated genes in T_FH_ cells were apparently enriched in T-cell proliferation and differentiation (Fig. 4b). Then we selected a T_FH_ cell and a GC T_FH_ cell signature gene set^11^ for gene set enrichment analysis (GSEA). Both the T_FH_ lineage gene set and the GC T_FH_-associated gene set were enriched in wild-type T_FH_ cells, but not in METTL3-deficient T_FH_ cells (Fig. 4c). When applying GSEA to exhibit the signature genes of T_H_1 (ref. ^12^), T_H_2 (ref. ^34^), T_H_17 (ref. ^35^), and Treg^34^ cells, we found that all of which were enriched in METTL3-deficient T_FH_ cells (Supplementary Fig. 5b). Similarly, we also observed T_FH_, T_H_2, T_H_17, and Treg-related gene sets were positively enriched in METTL3-deficient T_H_1 cells (Supplementary Fig. 5c). These analyses indicated that loss of METTL3 leads to disordered gene profiles of both T_FH_ and T_H_1 transcription program.

We next validated expression changes of key T_FH_ genes and found that *Cxcr5*, *Bcl6*, *Pdcd1*, and *Icos* were significantly lower in METTL3-null T_FH_ cells than in wild-type cells (Fig. 4e). In addition, the expression of *Tcf7* was lower in METTL3-deficient T_FH_ cells than in wild-type T_FH_ cells (Fig. 4e). Among the genes with low expression, *Tcf7* was of interest because of its role in Tfh cell differentiation, so we also performed GSEA analysis of a gene set containing TCF-1-activated genes in T_FH_ cells^13^. Interestingly, remarkable enrichment was exhibited in wild-type T_FH_ cells, whereas the TCF-1-suppressed gene set^13^ was notably enriched in METTL3-deficient cells, which suggested that METTL3 and TCF-1 share a common subset of target genes in T_FH_ cells (Fig. 4d). Meanwhile, the expression of *Lef1* mRNA was also decreased in METTL3-deficient T_FH_ cells compared with wild-type cells (Fig. 4e). On the other hand, the expression of *Prdm1*, known as the antagonist of *Bcl6*, and *Gzmb* was higher in METTL3-null T_FH_ cells than that in wild-type T_FH_ cells (Fig. 4e). The expression levels of *Bcl2l11* and *Bax*, both known as pro-apoptotic regulators^36,37^, were significantly elevated in METTL3-null T_FH_ cells compared with those in the wild-type T_FH_ cells (Fig. 4e), which corresponded to accelerated apoptosis in *Mettl3*^fl/fl^*Cd4*-Cre T_FH_ cells (Supplementary Fig. 4g, h). In addition, the transcripts encoding other essential transcription factors, receptors and ligands for T_FH_ differentiation, including *Batf*, *Slamf6*, and *Il6ra*, were downregulated in *Mettl3*^fl/fl^*Cd4*-Cre T_FH_ cells compared with those in wild-type cells (Fig. 4e). Besides, we observed elevated expression of *Socs1* mRNA, a known target of METTL3^25,26^, in *Mettl3*^fl/fl^*Cd4*-Cre T_FH_ cells (Fig. 4e). Collectively, these results suggested that METTL3 regulates T_FH_ differentiation program by activating T_FH_ program but suppressing T_H_1 lineage-associated genes.

### METTL3 promotes T_FH_ differentiation in an m^6^A catalytic activity-dependent manner

**Fig. 5:**
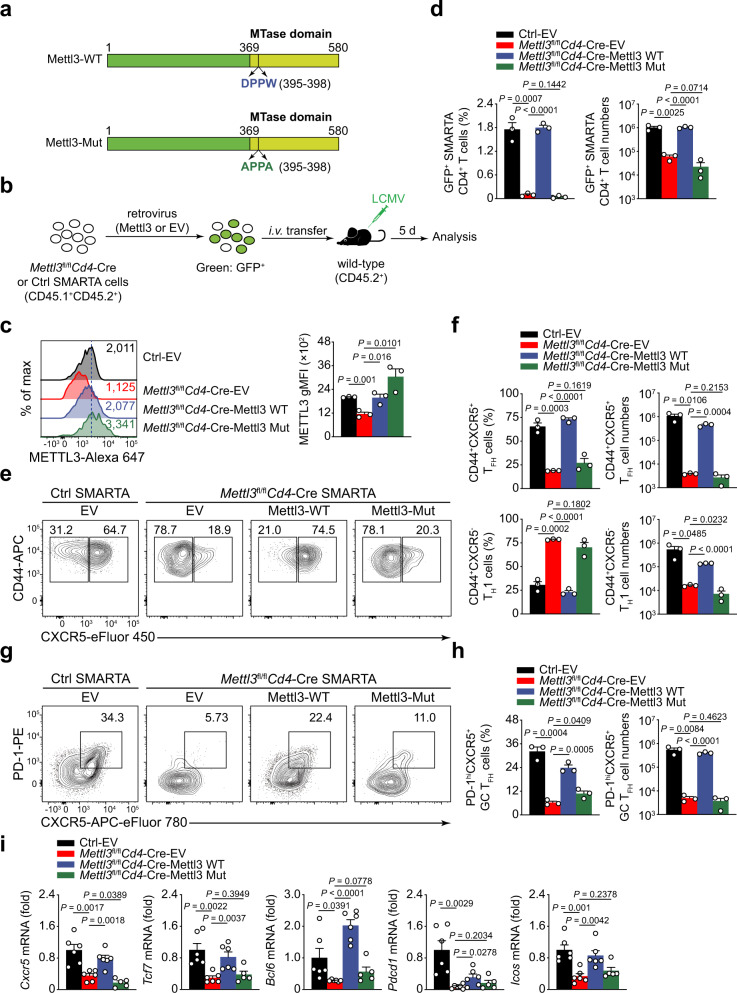
METTL3 promotes T_FH_ differentiation in an m^6^A catalytic activity-dependent manner. **a** Graphic representation of the wild-type (Mettl3-WT) and catalytic domain dead (Mettl3-Mut; DPPW to APPA) METTL3 constructs. **b** Scheme of retrovirus transduction experiment. SMARTA cells were transduced with indicated structures by using a retrovirus transduction system. Then, the transduced cells were adoptively transferred into congenic CD45.2^+^ wild-type mice followed by LCMV-Armstrong infection, and analyzed on day 5 post viral infection. **c** Flow cytometry analysis of METTL3 gMFI in GFP^+^CD4^+^ SMARTA cells from recipient mice. Quantitation of METTL3 gMFI is shown on the right (*n* = 3 per group). **d** Summary of the frequency and cell numbers of retrovirus-transduced SMARTA CD4^+^ T cells in host mice (*n* = 3 per group). **e**, **f** Flow cytometry analysis of CD44^+^CXCR5^+^ T_FH_ populations and CD44^+^CXCR5^–^ T_H_1 subsets gated on SMARTA GFP^+^CD4^+^ T cells from host mice adoptively transferred with empty vector (EV), Mettl3-WT, or Mettl3-Mut retrovirus-introduced SMARTA cells. Summary of the frequency and cell numbers of T_FH_ cells and T_H_1 cells are shown in **f** (*n* = 3 per group). **g**, **h** Flow cytometry analysis of PD-1^hi^CXCR5^+^ GC T_FH_ populations gated on SMARTA GFP^+^CD4^+^ T cells from host mice adoptively transferred with EV, Mettl3-WT, or Mettl3-Mut retrovirus-introduced SMARTA cells. Summary of the frequency and cell numbers of GC T_FH_ cells are shown in **h** (*n* = 3 per group). **i** Quantitative RT-PCR analysis of mRNA abundance of T_FH_ cell-related genes in CD44^+^CXCR5^+^ T_FH_ cells ectopically expressed with EV, Mettl3-WT, or Mettl3-Mut, relative expression was normalized to Ctrl cells transduced with EV retrovirus (*n* = 6 for Ctrl EV, *Mettl3*^fl/fl^*Cd4*-Cre-EV, and *Mettl3*^fl/fl^*Cd4*-Cre-Mettl3 WT group; *n* = 5 for *Mettl3*^fl/fl^*Cd4*-Cre-Mettl3 Mut group). Data are representative of two independent experiments. Error bars indicate standard error of the mean. *P* value was calculated by one-way ANOVA, followed by unpaired two-tailed Student’s *t* test for indicated pairwise comparisons.

m^6^A methylation is catalyzed by a multicomponent methyltransferase complex, while METTL3 functions as the predominant catalytic subunit^15,16^. Next, we analyzed whether METTL3-mediated T_FH_ differentiation is dependent on its m^6^A methyltransferase activity. To achieve this goal, we generated wild-type METTL3 (Mettl3-WT) and catalytic domain mutant METTL3 (D395A and W398A; Mettl3-Mut) constructs^15^ (Fig. 5a). These METTL3 proteins were then expressed in *Mettl3*^fl/fl^*Cd4*-Cre SMARTA CD4^+^ T cells with a retrovirus transduction system (Fig. 5b). On day 5 post infection, forced expression of Mettl3-WT and Mettl3-Mut both resulted in elevated METTL3 expression in *Mettl3*^fl/fl^*Cd4*-Cre SMARTA cells compared with the empty-vector (EV) retrovirus (Fig. 5c). EV retrovirus-infected *Mettl3*^fl/fl^*Cd4*-Cre SMARTA CD4^+^ T cells exhibited reduced expansion than EV retrovirus-infected Ctrl cells, which could be restored by overexpression of Mettl3-WT but not Mettl3-Mut (Fig. 5d). Moreover, EV retrovirus-infected *Mettl3*^fl/fl^*Cd4*-Cre SMARTA CD4^+^ T cells remained defective in the generation of CD44^+^CXCR5^+^ T_FH_ cells, while Mettl3-WT but not Mettl3-Mut retrovirus promoted differentiation of *Mettl3*^fl/fl^*Cd4*-Cre SMARTA CD4^+^ T cells into a T_FH_ fate (Fig. 5e). In addition, forced expression of Mettl3-WT could restore both the cell numbers of *Mettl3*^fl/fl^*Cd4*-Cre T_FH_ cells and T_H_1 cells (Fig. 5f). Accordingly, ectopic expression of Mettl3-WT could largely rectify defective PD-1^hi^CXCR5^+^ GC T_FH_ cells in METTL3-deficient cells (Fig. 5g, h). We also observed that the expression of T_FH_ cell-related genes, including *Cxcr5*, *Tcf7*, *Bcl6*, *Pdcd1*, and *Icos*, were restored in *Mettl3*^fl/fl^*Cd4*-Cre cells with inducing Mettl3-WT expression (Fig. 5i). The data collectively indicated that the m^6^A-catalytic activity of METTL3 is necessary for T_FH_ differentiation.

### m^6^A modifies *Tcf7* mRNA in 3′ UTR to control its stability

**Fig. 6:**
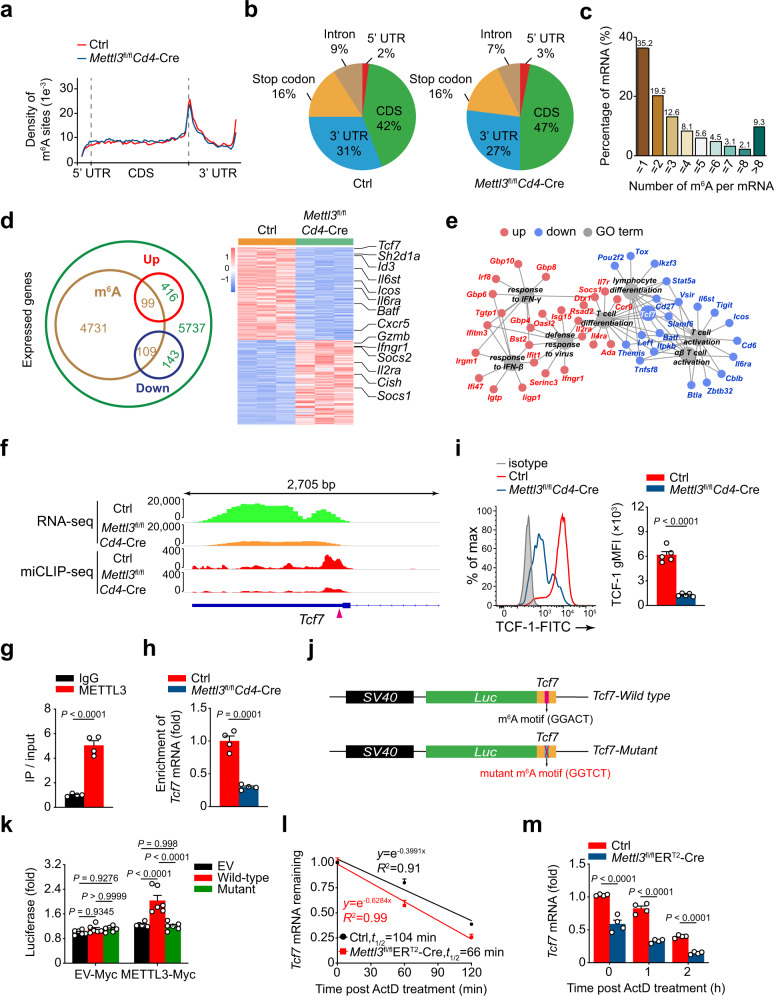
m^6^A modifies *Tcf7* mRNA to control its stability. **a** Metagene profiles of m^6^A site distribution along a normalized transcript containing three rescaled non-overlapping segments: 5′ UTR, CDS, and 3′ UTR in Ctrl and *Mettl3*^fl/fl^*Cd4*-Cre SMARTA CD4^+^ T cells. **b** Pie chart showing the distribution of m^6^A sites in five regions of Ctrl and *Mettl3*^fl/fl^*Cd4*-Cre SMARTA CD4^+^ T cells. **c** Bar chart depicting percentage of mRNAs with different internal m^6^A abundance. **d** Venn diagram showing the overlapping differentially expressed genes from RNA-seq and m^6^A-modified transcripts from m^6^A-miCLIP-SMARTer-seq. Heatmap of 208 differentially expressed genes with m^6^A modification is shown on the right. **e** Representative GO terms of the biological process categories enriched in differentially expressed transcripts with m^6^A peaks (Red: upregulated genes; blue: downregulated genes; gray: GO terms). **f** Integrative Genomics Viewer (IGV) tracks displaying RNA-seq (top panel) and m^6^A-miCLIP-SMARTer-seq (bottom panel) reads distribution of *Tcf7* gene. The high-confidence m^6^A site is marked as a triangle. **g** RIP-qPCR analysis showing enrichment of METTL3 on *Tcf7* mRNA in SMARTA CD4^+^ T cells. The *Tcf7* mRNA enrichment is presented as IP/input and normalized to IgG group (*n* = 4 per group). **h** m^6^A-RIP-qPCR analysis of m^6^A enrichment on *Tcf7* mRNA of Ctrl and *Mettl3*^fl/fl^*Cd4*-Cre SMARTA CD4^+^ T cells (*n* = 4 per group). **i** Flow cytometry analysis of expression level of TCF-1 on T_FH_ cells on 8 *dpi*. Quantification of gMFI of TCF-1 is shown on the right (*n* = 5 per group). **j** Constructions of plasmids with wild-type or m^6^A site mutant in *Tcf7* 3′ UTR. **k** Luciferase reporter assay. Results were normalized to the luciferase activity of cells co-transfected with the pGL4.23 empty plasmid and EV-Myc plasmid (*n* = 6 per group). **l** RNA decay assay. The half-live of *Tcf7* mRNA was detected by quantitative RT-PCR. The remaining mRNAs were normalized to *t* = 0 (*n* = 4 per group). **m** Quantitative RT-PCR analysis of *Tcf7* mRNA abundance as in **l**, relative expression was normalized to *t* = 0 of Ctrl cells (*n* = 4 per group). Data are from one experiment with duplicate (**a**–**e**) or representative of at least three independent experiments (**i**–**h**, **k**–**m**). Error bars indicate standard error of the mean. *P* value was calculated by unpaired two-tailed Student’s *t* test (**g**–**i**) or two-way ANOVA coupled with multiple comparisons (**k**, **m**).

To examine how m^6^A methylation modulates T_FH_ transcriptional program, we performed m^6^A-miCLIP-SMARTer-seq to map global m^6^A landscape in Ctrl or *Mettl3*^fl/fl^*Cd4*-Cre SMARTA CD4^+^ T cells primed in vivo. Bioinformatic analysis revealed that m^6^A peaks were significantly abundant in 3′ UTR and near the stop codon of mRNAs (Fig. 6a, b). Approximately 45.3% methylated mRNAs contained three or more peaks (Fig. 6c). Biological duplicates of m^6^A-miCLIP-SMARTer-seq yielded about 4939 transcripts (called ‘m^6^A-modified transcripts’ hereafter; Fig. 6d). By stratifying m^6^A-modified transcripts with differentially expressed genes, we found that 208 differentially expressed transcripts were potentially regulated by m^6^A methylation (Fig. 6d). GO term analysis showed these transcripts were enriched for functions related to defense response to the virus, T-cell activation and differentiation (Fig. 6e). Among these potential m^6^A methylated target genes, a set of T_FH_ cell-relevant genes were directly marked by m^6^A, such as *Cxcr5*, *Icos*, *Il6ra*, and *Il6st* (Supplementary Fig. 6a). Interestingly, the 3′ UTR of *Tcf7* mRNA had highly enriched m^6^A peaks, whereas *Bcl6* and *Prdm1* mRNAs were not tagged by m^6^A (Fig. 6f and Supplementary Fig. 6a). Accordingly, RNA immunoprecipitation (RIP) assay suggested METTL3 directly binds to *Tcf7* mRNA (Fig. 6g). The m^6^A-RIP-qPCR results further indicated *Tcf7* mRNA was tagged by m^6^A methylation and the m^6^A levels on *Tcf7* mRNA were substantially decreased in METTL3-deficient cells (Fig. 6f, h). These data jointly indicated *Tcf7* is a bona fide m^6^A target. Accordingly, both the *Tcf7* mRNA and the TCF-1 protein were significantly decreased in METTL3-deficient T_FH_ cells compared with those in wild-type T_FH_ cells (Fig. 4e and Fig. 6i). To further validate METTL3 regulation of *Tcf7* expression in an m^6^A-dependent manner, we identified a high-confidence m^6^A site (GGA_1011_CT, a conserved m^6^A methylation motif) in the *Tcf7* 3′ UTR region (Fig. 6f). We generated a minigene vector placing the *Tcf7* m^6^A site to the 3′ end of luciferase reporter cDNA. Then the ‘GGACT’ consensus motif in the *Tcf7* m^6^A site was mutated into ‘GGTCT’ to abrogate m^6^A modification (Fig. 6j). Wild-type *Tcf7* 3′ UTR exhibited increased luciferase activity compared to that of the empty vector (pGL4.23) in the presence of overexpressed METTL3; notably, mutation of *Tcf7* 3′ UTR almost completely compromised the increase (Fig. 6k). These results revealed that METTL3 regulates *Tcf7* mRNA levels via m^6^A modifications in the 3′ UTR region (m^6^A_1011_). Given m^6^A modification regulates gene expression by multiple approaches, including mRNA splicing, stability, and translation^17,38^, alternative splicing assay was applied and exhibited no significant difference in the alternative splicing of *Tcf7* mRNA between Ctrl and METTL3-deficient T_FH_ cells (Supplementary Fig. 6b). We then performed RNA decay assay via actinomycin D (ActD) treatment to detect the stability of *Tcf7* mRNA, and found that *Tcf7* mRNA exhibited substantially accelerated decline in METTL3-deficient cells compared with Ctrl cells at checkpoints after treatment (Fig. 6l, m). Taken together, these data suggested that METTL3 enhances *Tcf7* mRNA stability via catalyzing m^6^A methylation at its 3′ UTR, to ensure TCF-1 expression in promoting T_FH_ differentiation.

### Forced expression of TCF-1 restores defective T_FH_ differentiation in METTL3-deficient mice

**Fig. 7:**
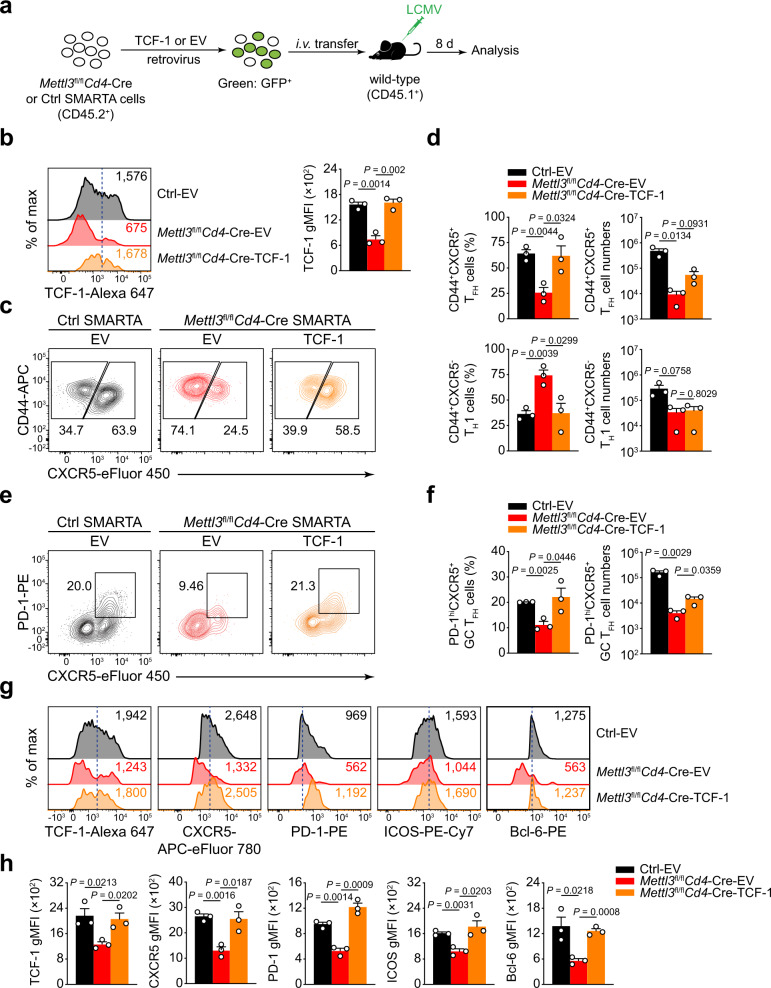
Enhanced TCF-1 expression rectifies defective T_FH_ differentiation in METTL3-null cell. **a** Scheme of TCF-1 rescue experiment. SMARTA cells were transduced with TCF-1 structure (full-length CDS of P45 isoform without 3′ UTR region) by using a retrovirus transduction system. Then, the transduced cells were adoptively transferred into congenic CD45.1^+^ wild-type mice followed by LCMV-Armstrong infection, and analyzed on day 8 post viral infection. **b** Flow cytometry analysis of TCF-1 gMFI in SMARTA GFP^+^CD4^+^ cells from recipient mice on 8 *dpi*. Quantitation of TCF-1 gMFI is shown on the right (*n* = 3 per group). **c**, **d** Flow cytometry analysis of CD44^+^CXCR5^+^ T_FH_ populations and CD44^+^CXCR5^–^ T_H_1 subsets gated on SMARTA GFP^+^CD4^+^ T cells from different host mice adoptively transferred with empty vector (EV) or TCF-1 retrovirus-introduced SMARTA cells at 8 days post infection. Summary of the frequency and cell numbers of T_FH_ cells and T_H_1 cells are shown in **d** (*n* = 3 per group). **e**, **f** Flow cytometry analysis of PD-1^hi^CXCR5^+^ GC T_FH_ populations gated on SMARTA GFP^+^CD4^+^ T cells from different host mice adoptively transferred with EV or TCF-1 retrovirus-introduced SMARTA cells. Summary of the frequency and cell numbers of GC T_FH_ cells are shown in **f** (*n* = 3 per group). **g**, **h** Flow cytometry analysis of gMFIs of TCF-1, CXCR5, PD-1, ICOS, and Bcl-6 on CD44^+^CXCR5^+^ T_FH_ cells transduced with EV or TCF-1 retrovirus. Quantification of the gMFIs is shown in **h** (*n* = 3 per group). Data are representative of three independent experiments. Error bars indicate standard error of the mean. *P* value was calculated by one-way ANOVA, followed by unpaired two-tailed Student’s *t* test for indicated pairwise comparisons.

To determine the functional link between METTL3 and *Tcf7* transcript stability in T_FH_ differentiation, we next examined the impact of ectopic expression of TCF-1 in METTL3-deficient cells. Upon transducing in vivo primed SMARTA CD4^+^ T cells with TCF-1 (full-length CDS of P45 isoform without 3′ untranslated region^39^) retrovirus (Fig. 7a), on day 8 post LCMV-Armstrong infection, we observed a significant increase in TCF-1 expression in METTL3-deficient SMARTA cells (Fig. 7b). We then analyzed T_FH_ populations from transduced SMARTA CD4^+^ T cells in recipient mice. Compared with Ctrl SMARTA CD4^+^ T cells infected with EV retrovirus, the EV-infected *Mettl3*^fl/fl^*Cd4*-Cre SMARTA CD4^+^ T cells exhibited defects in CD44^+^CXCR5^+^ T_FH_ differentiation; whereas TCF-1 retrovirus could largely rectify the ability of *Mettl3*^fl/fl^*Cd4*-Cre SMARTA CD4^+^ T cells to differentiate into T_FH_ cells (Fig. 7c). In addition, TCF-1 overexpression also elevated the cell numbers of *Mettl3*^fl/fl^*Cd4*-Cre T_FH_ cells, but not T_H_1 cells (Fig. 7d). Consistently, PD-1^hi^CXCR5^+^ GC T_FH_ cells could also be rescued with TCF-1 overexpression (Fig. 7e, f). Meanwhile, forced expression of TCF-1 could largely restore the expression levels of TCF-1, CXCR5, PD-1, ICOS, and Bcl-6 on *Mettl3*^fl/fl^*Cd4*-Cre T_FH_ cells (Fig. 7g, h). These data collectively demonstrated that METTL3 stabilizes *Tcf7* mRNA expression to promote T_FH_ differentiation.

## Discussion

*N*^6^-methyladenosine (m^6^A) accounts for the prevalent mRNA modifications and has recently emerged as a critical epitranscriptomic regulator to affect the translation and stability of the modified transcripts. Here, we showed that ablation of METTL3 leads to substantial defects in both T_FH_ and T_H_1 differentiation upon LCMV-Armstrong and KLH challenges. Based on our results, a more severe phenotype was exhibited in T_FH_ cells than that of T_H_1 cells from METTL3-deficient mice, which strongly supports the notion that the METTL3 acts as an intrinsic modulator of T_FH_ cells.

During acute viral infection, bifurcation of effector CD4^+^ T cells into T_FH_ cells or T_H_1 cells can be observed as early as the second to third division^40^. A recent report demonstrated that METTL3-null CD4^+^ T cells remained naive state and exhibited defective proliferation when tested both in vivo and in vitro^25^. Using an adoptive transfer model, we found that METTL3-deficient CD4^+^ T cells showed a delayed differentiation and a slower proliferation upon acute viral infection, although they were activated normally as shown by expression of the surface markers via both in vivo and in vitro assay. Meanwhile, at 72 h post infection, the activated CD4^+^ T cells were mostly in the fifth and sixth divisions, whereas METTL3-deficient CD4^+^ T cells were dominant in third and fourth divisions. These METTL3-deficient CD4^+^ T cells also showed defects in differentiation into T_FH_ cells and exhibited elevated apoptosis. Further, METTL3 deficiency also resulted in a decreased expression of T_FH_-defining genes in these nascent T_FH_ cells. Strikingly, the abundances of *Tcf7* and *Bcl6* mRNAs, both essential for the early commitment of T_FH_ cells^41^, were profoundly decreased in early METTL3-null T_FH_ cells. Correspondingly, the protein levels of TCF-1 and Bcl-6 were also notably reduced. These findings revealed that METTL3 is required for early T_FH_ commitment, proliferation, and survival by maintaining the key T_FH_ gene expression.

As an RNA binding protein, METTL3 is the essential catalytic component of the conserved heterodimeric m^6^A writer complex^15^. Does METTL3-mediated T_FH_ differentiation also directly depend on its m^6^A catalytic activity? Our results showed that forced expression of METTL3 with a mutated catalytic domain failed to rectify the defects in T_FH_ differentiation of *Mettl3*^fl/fl^*Cd4*-Cre CD4^+^ T cells, while wild-type METTL3 did. We thus concluded that METTL3 promotes T_FH_ generation in an m^6^A catalytic activity-dependent manner. Giving m^6^A methylation is essential for gene expression regulation^42^, we hence focused on the methylation status of those differentially expressed genes. The expression patterns of a series of T_FH_-defining genes were altered at both early and mature stages, including *Tcf7*, *Cxcr5*, *Bcl6*, *Pdcd1*, and *Icos*. Through m^6^A-miCLIP-SMARTer-seq, we found *Tcf7* mRNA was tagged by m^6^A in the 3′ UTR, whereas the other key T_FH_ cell regulator *Bcl6* mRNA was not modified. Further study indicated that METTL3-mediated m^6^A modification regulates the stability of *Tcf7* mRNA, ultimately maintains TCF-1 level. Moreover, forced expression of TCF-1 could restore the T_FH_ differentiation in the absence of METTL3. These data collectively suggested that METTL3 directly modulates the *Tcf7* expression level in an m^6^A-dependent manner to promote T_FH_ differentiation.

The functional outcome of m^6^A modification is determined by an expanding list of m^6^A reader proteins in a cell type and cellular context-dependent fashion^14,43^. Although early evidence demonstrated that m^6^A deposition destabilizes mRNAs, resulting in their faster decay, a recent study has reported that the insulin-like growth factor 2 mRNA binding proteins (IGF2BP1-3), as a class of distinct m^6^A readers, promote m^6^A-modified mRNA stability and translation^20^. Similarly, a more recent study reported Prrc2a as a novel m^6^A reader that regulates oligodendrocyte specification and myelination by stabilizing target mRNA^44^. Our data also indicated m^6^A modification in the 3′ UTR of *Tcf7* enhances its mRNA stability to promote T_FH_ differentiation. However, the currently known IGF2BPs family readers are expressed at extremely low levels to detect in CD4^+^ T cells (Supplementary Fig. 8), implying the possibility of other unidentified proteins might be responsible for deciphering the m^6^A-modified transcripts in T_FH_ cells. Therefore, it will be of great interest to identify new RNA-binding proteins virtually involved in T_FH_ cells via post-transcriptional networks in future studies.

In METTL3-deficient cells, destabilization of *Tcf7* mRNA impacted T_FH_ cells at least in two major aspects. The first aspect is that loss of TCF-1 expression results in massive apoptosis in T cell^45^, which is coordinated with the decreased T_FH_ cell numbers in the absence of METTL3. The other point lies in that TCF-1 directly regulates *Bcl6* mRNA expression^11–13^, and two of them thus collectively affect T_FH_ differentiation. This post-transcriptional regulation of *Tcf7* mRNA represents a distinct mechanism that drives T_FH_ differentiation. It should be noted that other post-transcriptional regulators such as RNA-binding proteins and miRNAs also contribute to modulating T_FH_ differentiation program^46–48^. Hence, exploring T_FH_ fate determination on the layer of post-transcriptional level might be a fruitful effort in future investigations.

A recent study referred that induced GAPDH protein by VHL deficiency reduced *Icos* expression through METTL3/METTL14-catalyzed m^6^A modification on *Icos* mRNA, implying that elevated m^6^A modification on *Icos* mRNA in VHL-deficient cells decreases *Icos* expression which is associated with attenuated T_FH_ differentiation^49^. By analyzing high-throughput data, we also observed m^6^A modification in the 3′ UTR of *Icos* mRNA, and the m^6^A level on *Icos* mRNA was decreased in the absence of METTL3. However, we found both the mRNA and protein level of ICOS were blunted in METTL3-deficient T_FH_ cells, indicating that loss of m^6^A modification impairs *Icos* expression. In addition, Zhu et al. reported that knockdown of METTL3 expression with short hairpin RNA (shRNA) in CD4^+^ T cells could promote T_FH_ differentiation^49^, which differs in phenotypes from our genetic knockdown mice model. The varies may be contributed by the distinct experimental approaches and the divergent viewpoints from two studies also reflect the complex regulatory mechanism of m^6^A modification, which needs to be further disclosed.

**Fig. 8:**
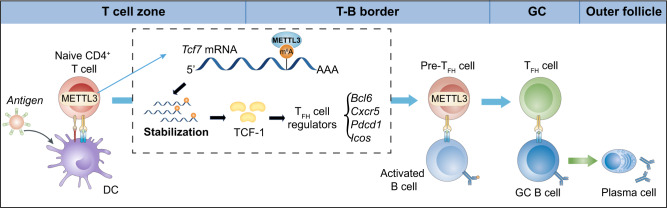
Proposed model for m^6^A modification in promoting T_FH_ differentiation. During acute infection, METTL3-sufficient CD4^+^ T cells were activated. With m^6^A machinery, *Tcf7* mRNA was m^6^A modified and stabilized, allowing normal production of TCF-1 protein. TCF-1 in turn regulates expressions of T_FH_ cell regulators, which ultimately program T_FH_ commitment, proliferation, survival, and functional maturation.

In summary, our study uncovers a critical role of METTL3-dependent m^6^A methylation in directing T_FH_ lineage differentiation. Conditional ablation of m^6^A ‘writer’ METTL3 in CD4^+^ T cells intrinsically impaired the T_FH_ differentiation, proliferation, and survival. Consequently, the GC reactions were significantly compromised in METTL3-deficient mice in response to acute viral infection. Our data indicated that METTL3 directs m^6^A modification in 3′ UTR of *Tcf7* mRNA to stabilize the transcript and hence sustain TCF-1 protein expression (Fig. 8). Thus, m^6^A functions as an important modulator of the METTL3-TCF-1 axis to initiate and secure the differentiation of T_FH_ cells post-transcriptionally.

## Methods

### Mice

*Mettl3*^fl/fl^ mice were kindly provided by Drs. Qi Zhou and Wei Li (Institute of Zoology, Chinese Academy of Sciences). SMARTA mice^50^ (expressing MHC II I-A^b^-restricted TCR specific for LCMV glycoprotein amino acids 66–77) were generously provided by Dr. Rafi Ahmed (Emory University). *Cd4*-Cre, ER^T2^-Cre, and C57BL/6J (CD45.2 and CD45.1) mice were purchased from the Jackson Laboratory. All mouse strains used in this study are on a fully C57BL/6J background. All mice were kept in group housing (3–5 mice per cage) in a specific pathogen-free facility with controlled environmental conditions of humidity (50 ± 10%), lighting (a 12-h light/dark cycle), and temperature (21 ± 1 °C) at China Agricultural University. All animal experiments were performed in accordance with the protocol of the Institutional Animal Care and Use Committee of China Agricultural University.

### LCMV infection

LCMV-Armstrong strain was grown in BHK-21 cells and titers were determined as described before^51^. *Mettl3*^fl/fl^*Cd4*-Cre mice and their wild-type littermates were intraperitoneally infected with 2 × 10^5^ plaque-forming units (pfu) LCMV-Armstrong strain. In adoptive transfer experiments, recipient mice were infected 1 day after cell transfer. For bone marrow chimeric mice, LCMV infection was performed after 8 weeks reconstitution.

### Immunization

*Mettl3*^fl/fl^*Cd4*-Cre mice and their wild-type littermates were intraperitoneally immunized with 100 μg of KLH (Sigma-Aldrich) emulsified in CFA (Sigma-Aldrich). Eight days later, splenic T cells were analyzed.

### Flow cytometry and antibodies

Single-cell suspensions of spleens were used for flow cytometry analysis or cell sorting. Surface staining was performed in PBS containing 1% FBS. The antibodies and reagents used for flow cytometry staining are listed as: anti-CD19 (1D3; 1:100), anti-CD25 (PC61.5; 1:100), anti-CD4 (RM4-5; 1:100), anti-CD44 (IM7; 1:100), anti-CD45.1 (A20; 1:100), anti-CD45.2 (104; 1:100), anti-CD62L (MEL-14; 1:100), anti-CD69 (H1.2F3; 1:100), anti-CD8a (53-6.7; 1:100), anti-B220 (RA3-6B2; 1:100), anti-GITR (DTA-1; 1:100), anti-GL7 (GL7; 1:100), anti-PD-1 (J43; 1:100), anti-TCR Vα2 (B20.1; 1:100) (from Thermo Fisher Scientific); anti-CD138 (281-2; 1:100), anti-Fas (Jo2; 1:100) (from BD Biosciences); anti-SLAM (TC15-12F12.2; 1:100), anti-ICOS (C398.4A; 1:100) (from BioLegend), and peanut agglutinin (PNA; Cat. no. FL-1071; 1:500; Vector laboratories). CXCR5 staining was performed with a three steps staining protocol as described before^52^. Briefly, single-cell suspensions were first stained with purified anti-CXCR5 (2G8; 1:100; BD Biosciences) for 1 h, followed by biotin-conjugated goat anti-rat IgG (Cat. no. 111-066-144; 1:1,000; Jackson ImmunoResearch) for 30 min, and then by APC-eFluor 780-, or eFluor 450-labeled streptavidin (1:500; Thermo Fisher Scientific) at 4 °C for 30 min in PBS supplemented with 2% normal mouse serum (Cat. no. 015-000-120; Jackson ImmunoResearch), 2% FCS, and 0.5% BSA. For detection of cytokines, the splenocytes from KLH-immunized mice were cultured in vitro for 5 h in 2 μg/mL of PMA (Cat. no. P8139; Sigma-Aldrich), 2 μg/mL of Ionomycin (Cat. no. I0634; Sigma-Aldrich), GolgiStop (BD Biosciences), and GolgiPlug (BD Biosciences). Intracellular staining for cytokines was performed with monoclonal antibody against to IL-4 (11B11; 1:100; Thermo Fisher Scientific) and IL-17a (TC11-18H10; 1:100; BD Biosciences), using the Fixation/Permeabilization buffer kit (BD Biosciences). For intracellular staining of Bcl-6 (K112-91; 1:20; BD Biosciences), Foxp3 (FJK-16s; 1:100; Thermo Fisher Scientific), GATA3 (TWAJ; 1:100; Thermo Fisher Scientific), RORγt (AFKJS-9; 1:100; Thermo Fisher Scientific), TCF-1 (C63D9; 1:100; Cell Signaling Technology), and METTL3 (Cat. no. ab195352; 1:100; Abcam), Foxp3/Transcription Factor Staining Buffer Set (Thermo Fisher Scientific) was used following the manufacturer’s instructions. Active Caspase-3 was detected using the CaspGLOW™ Fluorescein Active Caspase-3 Staining Kit (Cat. no. 88-7004-42; 1:200; Thermo Fisher Scientific). All data were collected on a FACSVerse (BD Biosciences) with FACSSuite software (v1.0.5) or an LSRFortessa (BD Biosciences) with FACSDiva software (v8.0.2) and were analyzed with FlowJo software (v10; Treestar). The gating strategies for flow cytometry data analysis are illustrated in Supplementary Fig. 7.

### Adoptive transfer

To characterization of cell division at early T_FH_ differentiation, SMARTA CD4^+^ T cells were labeled with 5 μM of CTV (Invitrogen), and 2 × 10^6^ of labeled Vα2^+^ SMARTA CD4^+^ cells were transferred followed by intravenously infected with 2 × 10^6^ pfu of LCMV-Armstrong. To investigate the intrinsic effects with adoptive transfer model, 5 × 10^6^ wild-type SMARTA cells were transferred into Ctrl and *Mettl3*^fl/fl^*Cd4*-Cre host mice, followed by 2 × 10^5^ pfu LCMV-Armstrong infection intraperitoneally.

### Bone marrow chimeric mice

To generate bone marrow chimeric mice, lethally irradiated B6.SJL (CD45.1^+^) mice were transferred intravenously with a 1:1 mixture of 2.5 × 10^6^ *Mettl3*^fl/fl^*Cd4*-Cre (CD45.2^+^) and 2.5 × 10^6^ B6.SJL (CD45.1^+^) bone marrow cells. After 8 weeks reconstitution, recipient mice were infected with LCMV-Armstrong strain.

### ELISA

Analysis of the LCMV-specific antibody in serum was performed as previously described^53^. Briefly, lysates of LCMV-infected BHK-21 cells were used as substrate and LCMV-specific antibody (IgG) was titrated in serial dilutions of serum using HPR-conjugated goat-anti mouse IgG antibodies (Cat. no. A90-131P-39; 1:5,000; Bethyl laboratories).

### Immunofluorescence staining

Tissue specimens submerged in OCT compound were quickly frozen in liquid nitrogen and cut into 10 μm thickness. Frozen tissue sections were then fixed in cold acetone for 30 min at −20 °C, blocked with 1% BSA and Fc-blocker (2.4G2; 1:100; BD Biosciences) in PBS. The tissue sections were then stained with biotinylated PNA (Cat. no. BA-0074; 1:20; Vector laboratories), followed by staining with BV510-labeled anti-CD4 (RM4-5; 1:100; BD Biosciences), APC-labeled anti-IgD (11-26c; 1:100; Thermo Fisher Scientific), and AF488-conjugated streptavidin (1:500; Invitrogen). Then the slides were washed at least three times with PBS. Coverslips were mounted on slides using an antifade kit (Beyotime Biotechnology) and then examined using an Andor Dragonfly confocal microscope. The images were processed with Imaris (v8.1; Bitplane) and Image J (v1.52 g; NIH).

### Retroviral vectors, transduction, and cell transfer

*Tcf7* (full-length CDS region of P45 isoform without 3′ UTR region^39^) and *Mettl3* (wild-type and catalytic domain dead) coding sequences were amplified and cloned into the pMIG-R1 vector (MSCV-IRES-GFP). Retrovirus was packaged by transfection of HEK293T cells (Cat. no. CRL-3216; ATCC) with the retroviral vectors along with pCL^eco^ plasmid. SMARTA cells were activated in vivo by injection of 200 μg of LCMV GP61-80 (GLNGPDIYKGVYQFKSVEFD) peptide into *Mettl3*^fl/fl^*Cd4*-Cre SMARTA mice or their littermate wild-type transgenic mice. After 16~18 h, activated SMARTA cells were isolated, purified, and ‘spin-infected’ for 120 min at 37 °C by centrifugation (1000 × *g*) with freshly harvested retroviral supernatants supplemented with 8 μg/mL of polybrene (Sigma-Aldrich), then cultured overnight in the presence of 20 ng/mL of IL-2 (Peprotech), and 250 nM of LCMV GP61-80. The spinofection was repeated the next day, and a total of 0.5~1 × 10^6^ retroviral infected SMARTA CD4^+^ T cells were then adoptively transferred into recipient mice, followed by infection of the hosts with 2 × 10^5^ pfu LCMV-Armstrong within 24 h.

### RNA-seq and data analysis

For isolation of T_H_1 cells and T_FH_ cells, splenocytes from *Mettl3*^fl/fl^*Cd4*-Cre mice and their control littermates on day 8 post viral infection were subjected to depletion of cells positive for lineages markers by using biotin-conjugated antibodies (anti-B220 (RA3-6B2; 1:100), anti-CD8 (53-6.7; 1:100), anti-Gr.1 (RB6-8C5; 1:100), anti-CD11b (M1/70; 1:100), anti-CD11c (N418; 1:100), anti-TER119 (TER-119; 1:100), and anti-CD49b (DX5; 1:100); all from Thermo Fisher Scientific) coupled to Dynabeads M-280 Streptavidin (Invitrogen), followed by surface stained. CD44^+^SLAM^hi^ T_H_1 cells and CD44^+^SLAM^lo^ T_FH_ cells were sorted with a FACSAria II cell sorter (BD Biosciences) with FACSDiva software (v7.0) and subsequently lysed with TRIzol Reagent (Life technologies). Total RNAs were extracted and then subjected to Annoroad (Beijing, China) for library construction and RNA sequencing. The qualities of clean reads were assessed by FastQC (v0.11.5). Then the reads were mapped to mouse genome mm10 (version M17) using TopHat (v2.1.1). The read counts of all genes were estimated by HTseq (v0.6.1) and differentially expressed genes were identified by DESeq2 (v1.18.1). TPM, FPKM, and RPKM were calculated, and upregulated or downregulated genes in *Mettl3*^fl/fl^*Cd4*-Cre T_FH_ or T_H_1 cells were identified by expression changes ≥ 2-fold and FDR < 0.01.

### Quantitative RT-PCR

Total RNAs were extracted from sorted cells using RNeasy Mini Kit (Qiagen) followed by cDNA synthesis with FastQuant RT Kit (Tiangen). Quantitative RT-PCR was carried out with SuperReal PreMix Plus SYBR Green (Tiangen) on a CFX96 Connect^TM^ Real-Time System (Bio-Rad). Results were processed by Microsoft Excel and then were normalized to the expression of *Hprt1* transcripts. Fold differences in expression levels were calculated according to the 2^−ΔΔCT^ method. All primers used are listed in Supplementary Table 1.

### Gene set enrichment analysis

GSEA was performed with GSEA desktop software (v4.1.0) from the Broad Institute. The T_FH_ gene set^11^, GC T_FH_ gene set^11^, T_H_1 gene set^12^, T_H_2 gene set^34^, T_H_17 gene set^35^, and Treg gene set^34^ have been described before. The ‘TCF1-activated genes in T_FH_ cells’ gene set contains 569 genes that are downregulated by ≥1.5-fold in *Tcf7*^fl/fl^*Cd4*-Cre T_FH_ cells; The ‘TCF1-suppressed genes in T_FH_ cells’ gene set contains 513 genes that are upregulated by ≥1.5-fold in *Tcf7*^fl/fl^*Cd4*-Cre T_FH_ cells (GSE65693).

### Luciferase reporter assay

*Tcf7* 3′ UTR segment (~200 nt) was amplified from a mouse double-positive (DP) thymocytes cDNA library by PCR and inserted into the pGL4.23 vector (Promega) by using XbaI and FseI restriction sites. The *Tcf7* 3′ UTR mutation plasmids were generations from site-directed mutagenesis. All plasmids and mutations were verified by sequencing. HEK293T cells were seeded into 24-well plates in triplicate to allow 80% confluency in the next day. A total of 200 ng of reporter plasmids (Fluc) and 20 ng of Renilla luciferase (Rluc) control plasmids (pRL-TK) were co-transfected using Lipofectamine 2000 reagent (Invitrogen) under METTL3 overexpressing. Fluc and Rluc activities were measured 24 h later with the Dual-Luciferase Reporter Assay System (Promega) according to the manufacturer’s instructions. The relative luciferase activity was calculated by dividing Fluc by Rluc and normalized to pGL4.23 empty vector for each assay.

### RNA decay assay

CD4^+^ T cells were purified from *Mettl3*^fl/fl^ER^T2^-Cre mice and their control wild-type mice as described above. 5 × 10^5^ purified CD4^+^ T cells in RPMI 1640 medium supplemented with 10% fetal bovine serum, 20 ng/mL of IL-2, 10 ng/mL of IL-7, and 5 μM of 4-Hydroxytamoxifen (Sigma-Aldrich) were seeded into 48-well plates. After 48 h, actinomycin D (MedChemExpress) was added to a final concentration of 5 μM, and cells were harvested at *t* = 0, 1, 2 h after actinomycin D treatment. Total RNAs were extracted and subjected to RT-qPCR analysis. Results were processed by Microsoft Excel and then normalized to the expression of *Gapdh* transcript. Fold differences in expression levels were calculated according to the 2^–ΔΔCT^ method. All primers used are listed in Supplementary Table 1.

### m^6^A-miCLIP-SMARTer-seq and data processing

Total RNAs from CD4^+^ SMARTA cells sorted from LCMV GP61-80-primed *Mettl3*^fl/fl^*Cd4*-Cre SMARTA or Ctrl SMARTA mice were extracted with TRIzol Reagent (Life Technologies). mRNAs were further isolated from total RNAs using Dynabeads mRNA purification kit (Ambion). The procedures of m^6^A-miCLIP-SMARTer-seq were according to the previously reported methods with some modifications^54^. Briefly, 100 ng of mRNAs were fragmented to ~100 nt by using the fragmentation reagent (Life Technologies) and incubated with 5 μg of specific antibody against m^6^A (Abcam) in 500 μL of immunoprecipitation buffer (50 mM Tris-HCl (pH 7.4), 100 mM NaCl, 0.05% NP-40) with gentle rotation at 4 °C for 2 h. The mixture was then transferred into a clear flat-bottom 96-well plate (Corning) on ice and irradiated three times with 0.15 J/cm^−2^ at 254 nm in a CL-1000 Ultraviolet Crosslinker (UVP). The irradiated mixture was then transferred to a new tube and incubated with 50 μL of pre-washed Dynabeads Protein A (Life Technologies) at 4 °C for 2 h. After extensive washing twice with high-salt wash buffer (50 mM Tris-HCl (pH 7.4), 1 M NaCl, 1 mM EDTA, 1% NP-40, 0.1% SDS) and twice with immunoprecipitation buffer, the mixture on beads was subjected to dephosphorylation with T4 PNK (NEB) for 20 min at 37 °C. After extensive washing, the RNA was eluted from the beads by proteinase K (Sigma-Aldrich) digestion at 55 °C for 1 h, followed by phenol-chloroform extraction and ethanol precipitation. The purified RNA was subjected to library construction using a SMARTer smRNA-Seq Kit for Illumina (Clontech Laboratories) according to the manufacturer’s instructions. Sequencing was carried out on an Illumina X-ten platform. m^6^A-miCLIP-SMARTer-seq data (paired-end) were analyzed as previously described^55^. Adapter sequences at the 3′ ends were removed first. R2 reads were then transformed to reverse complementary sequences and merged with R1 reads. PolyA tails from the library were trimmed by Cutadapt (v1.17) and fastq2collapse (v1.1.3) was used to remove duplicated reads; afterward, the barcodes of reads were removed. Low-quality bases were discarded, and only reads longer than 18 nt were retained. The remaining reads were mapped to the reference genome (mm10) using BWA (v0.7.17) with the parameter: -n 0.06. The mutation information was extracted and PCR duplicates according to BWA’s results were removed separately by parseAlignment.pl (–map-qual 1 –min-len 18) and tag2collapse.pl (-EM 30 –seq-error-model alignment). CIMS.pl was used to calculate the coverage (*n*) of the mutation site and transition number (*m*). Mutation sites with parameters, *m* ≥ 3, *k*/*m* ≥ 0.01, and *k*/*m* ≤ 0.5, were kept. The remaining sites within the RRACH motif sequence were considered as m^6^A.

### m^6^A-RIP-qPCR

Purified mRNAs of CD4^+^ SMARTA cells sorted from *Mettl3*^fl/fl^*Cd4*-Cre SMARTA or Ctrl SMARTA mice were prepared and fragmented into ~100 nt by RNA fragmentation reagents (Life Technologies). Immunoprecipitation was performed using anti-m^6^A antibody (Abcam) as described above. The enrichment of m^6^A was measured with quantitative RT-PCR. Primers for m^6^A-RIP-qPCR are listed in Supplementary Table 1.

### RIP-qPCR

5 × 10^6^ CD4^+^ SMARTA T cells were isolated and lysed with 1 mL cell lysis buffer (150 mM KCl, 10 mM HEPES (pH 7.6), 2 mM EDTA, 0.5% NP-40, 0.5 mM DTT, 1:100 proteinase inhibitor cocktail, and 0.4 U/µL RNasin) at 4 °C for 30 min. After centrifugation, the supernatant (10% of which was kept as input) was subjected to RNA immunoprecipitation with anti-METTL3 (Abcam) coupled with Dynabeads Protein A (Life Technologies). RNA was isolated from the beads and input samples for RT-qPCR. Primers for RIP-qPCR are listed in Supplementary Table 1.

### Statistical analysis

Statistical analysis was performed with Prism 8.0 (GraphPad). An unpaired two-tailed Student’s *t* test with a 95% confidence interval, one-way ANOVA, or two-way ANOVA analysis was used to calculate *P* values.

### Reporting summary

Further information on research design is available in the Nature Research Reporting Summary linked to this article.

## Supplementary information

Supplementary Information

Peer Review File

Reporting Summary

## Data Availability

RNA-seq and m^6^A-miCLIP-SMARTer-seq datasets have been deposited in Gene Expression Omnibus (GEO) under the accession number GSE129650. All data are available from the corresponding author upon reasonable request. Source data are provided with this paper.

## Source data

Source Data
