# Supplementary material for: METTL3-dependent m6A modification programs T follicular helper cell differentiation
Source: Nat Commun. 2021 Feb 26;12:1333. doi: 10.1038/s41467-021-21594-6 (PMC7910450; doi:10.1038/s41467-021-21594-6)
Supplement: Supplementary file 1 — Supplementary Information [file 41467_2021_21594_MOESM1_ESM.pdf]

Supplementary information

METTL3-dependent m^6A modification programs T follicular helper cell differentiation

Yingpeng Yao#, Ying Yang#, Wenhui Guo#, Lifan Xu#, Menghao You, Yi-Chang Zhang, Zhen Sun, Xiao Cui, Guotao Yu, Zhihong Qi, Jingjing Liu, Fang Wang, Juanjuan Liu, Tianyan Zhao, Lilin Ye*, Yun-Gui Yang*, Shuyang Yu*

# These authors contributed equally to this work.

* Correspondence: yelilinlcmv@tmmu.edu.cn (L.Y.), ygyang@big.ac.cn (Y.-G.Y.), ysy@cau.edu.cn (S.Y.).

Yao et al. Supplementary Figure 1

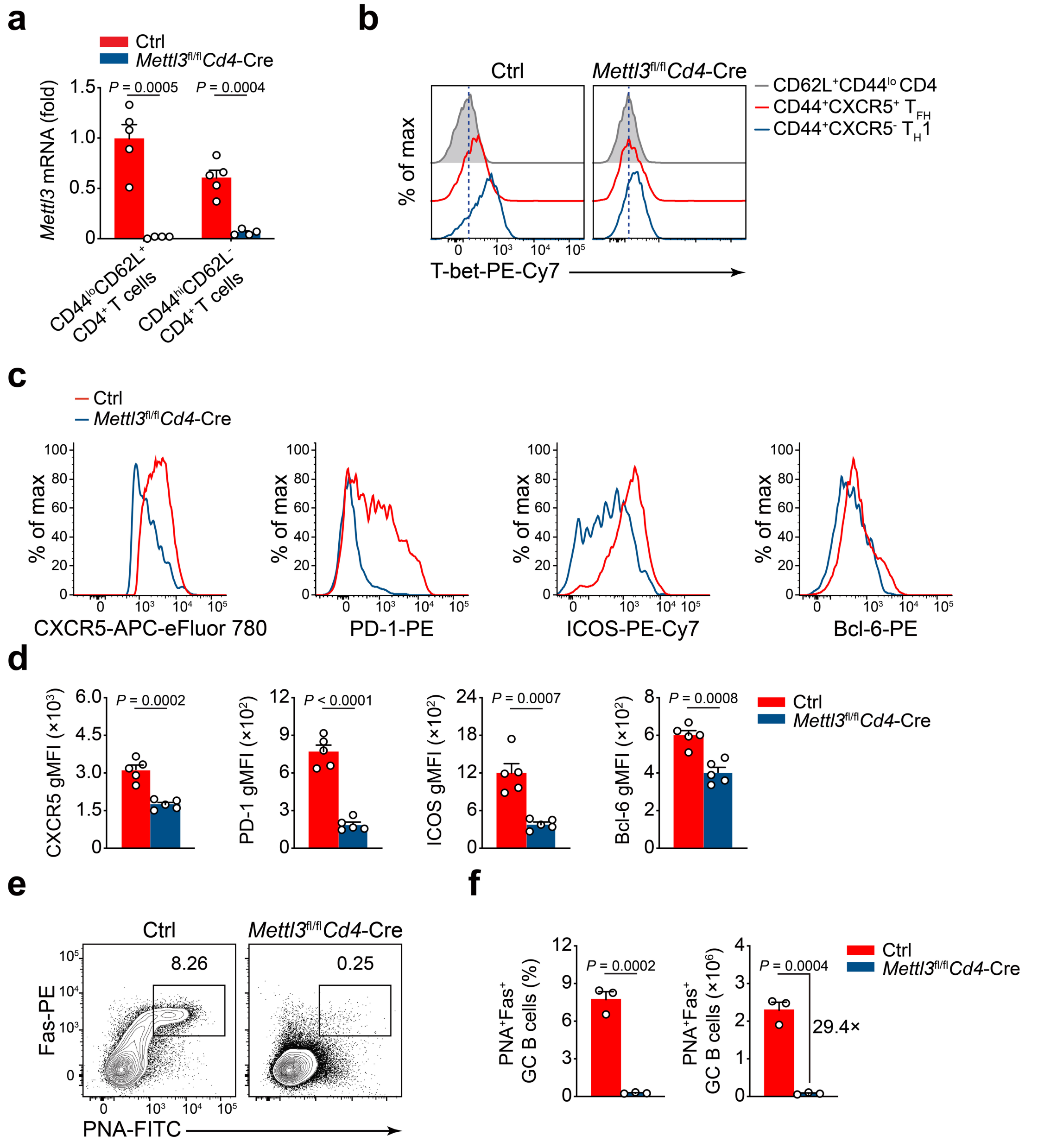

Supplementary Figure 1. METTL3 is critical for T$_{FH}$ differentiation and humoral immunity.

a Quantitative RT-PCR analysis of the abundance of *Mettl3* mRNA in CD44^{lo}CD62L^{+} CD4^{+} T cells (Naïve) and CD44^{hi}CD62L^{-}CD4^{+} T cells (Effector) from Ctrl and *Mettl3*$^{fl/fl}$*Cd4*-Cre mice, relative expression was normalized to Ctrl Naïve cells (n = 5 for Ctrl group, n = 4 for *Mettl3*$^{fl/fl}$*Cd4*-Cre group).

b Flow cytometry analysis of expression level of T-bet on $CD62L^{+}CD44^{lo}$ CD4 cells, $CD44^{+}CXCR5^{+}$ T_{FH} cells, and $CD44^{+}CXCR5^{-}$ $T_{H}1$ cells from Ctrl and *Mettl3*$^{fl/fl}$*Cd4*-Cre mice on 8 *dpi*.

c Flow cytometry analysis of expression levels of CXCR5, PD-1, ICOS, and Bcl-6 on $CD44^{+}CXCR5^{+}$ T_{FH} cells on 8 *dpi*.

d Quantification of the gMFIs of CXCR5, PD-1, ICOS, and Bcl-6 on $CD44^{+}CXCR5^{+}$ T_{FH} cells as described in **c** (n = 5 per group).

e, f Flow cytometry analysis of $PNA^{+}Fas^{+}$ GC B cells on 8 *dpi*. Summary of the frequency and cell numbers of GC B cells are shown in **f** (n = 3 per group).

Data are representative of two (**a**) or at least three (**b-f**) independent experiments. Error bars indicate standard error of the mean. P value was calculated by unpaired two-tailed Student's t-test.

Yao et al. Supplementary Figure 2

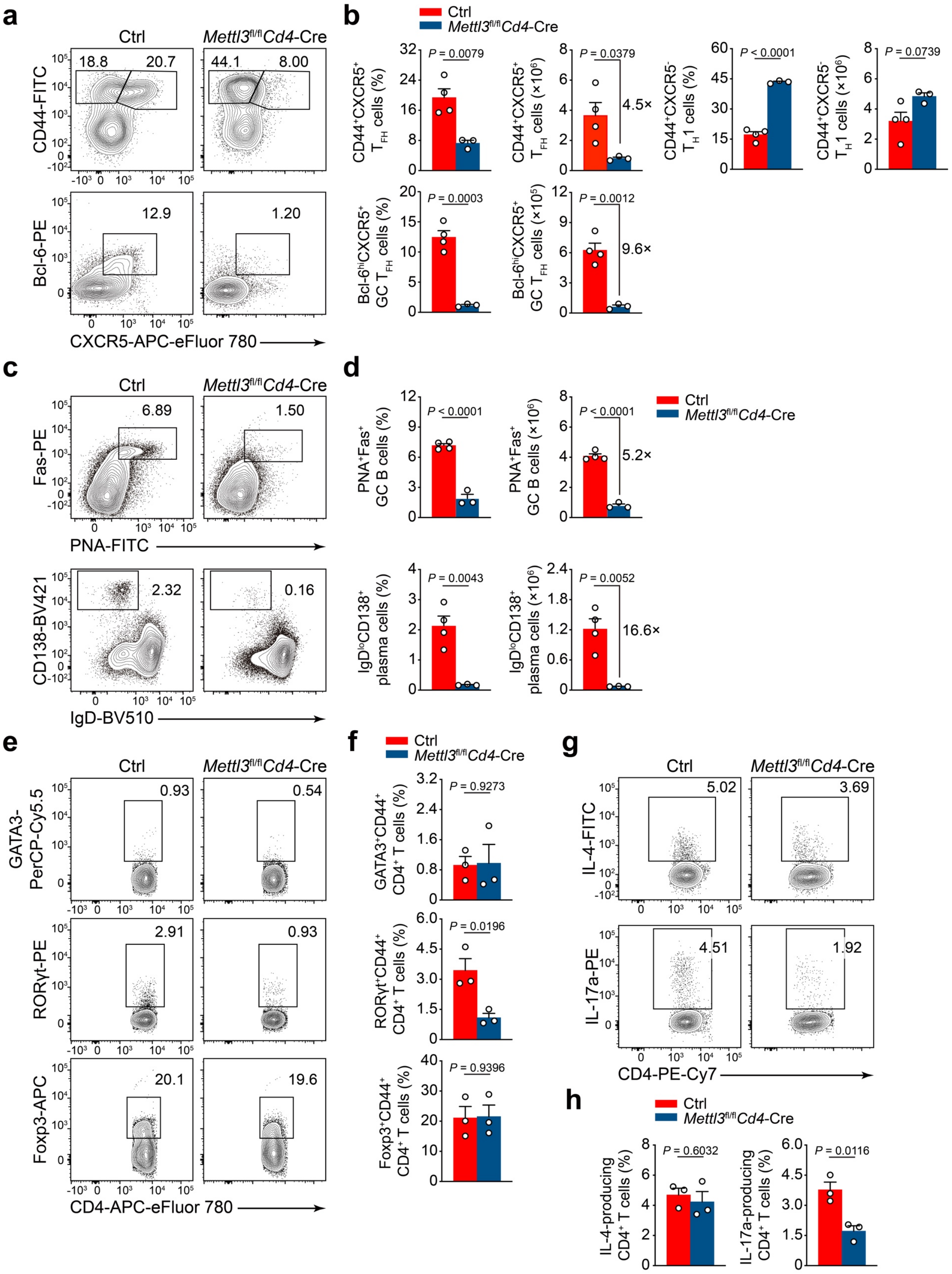

Supplementary Figure 2 METTL3 is essential for T_{FH} differentiation in KLH immunization model.

a, b Flow cytometry analysis of splenic $CD44^+CXCR5^+$ T_{FH} cells and $CD44^+CXCR5^-$ T_H1 cells gated on $CD4^+$ T cells (top panel), and Bcl-$6^{hi}CXCR5^+$ GC T_{FH} cells gated on $CD44^{hi}CD62L^{lo}CD4^+$ T cells (bottom panel) from Ctrl and *Mettl3*$^{fl/fl}$*Cd4*-Cre mice on day 8 post immunization. Summary of the frequency and cell numbers of T_{FH} cells, T_H1 cells, and GC T_{FH} cells are shown in **b** (n = 4 for Ctrl group, n = 3 for *Mettl3*$^{fl/fl}$*Cd4*-Cre group).

c, d Flow cytometry analysis of splenic Fas^+PNA^+ GC B cells (top panel) and $IgD^{lo}CD138^+$ plasma cells (bottom panel) on day 8 post immunization. Summary of the frequency and cell numbers of GC B cells and plasma cells are shown in **d** (n = 4 for Ctrl group, n = 3 for *Mettl3*$^{fl/fl}$*Cd4*-Cre group).

e, f Flow cytometry analysis of $GATA3^+$ (top panel), $ROR\gamma t^+$ (middle panel), and $Foxp3^+$ cells (bottom panel) gated on $CD44^{hi}CD62L^{lo}CD4^+$ T cells from Ctrl and *Mettl3*$^{fl/fl}$*Cd4*-Cre mice on day 8 post immunization. Summary of the frequency of indicated subsets is shown in **f** (n = 3 per group).

g, h Splenocytes from KLH-immunized Ctrl and *Mettl3*$^{fl/fl}$*Cd4*-Cre mice were restimulated with PMA and Ionomycin for 5 h at 37°C for detection of IL-4 and IL-17a expression by intracellular cytokine staining. Summary of the frequency of indicated subsets is shown in **h** (n = 3 per group).

Data are representative of two (**e-h**) or three (**a-d**) independent experiments. Error bars indicate standard error of the mean. *P* value was calculated by unpaired two-tailed Student's *t*-test.

Yao et al. Supplementary Figure 3

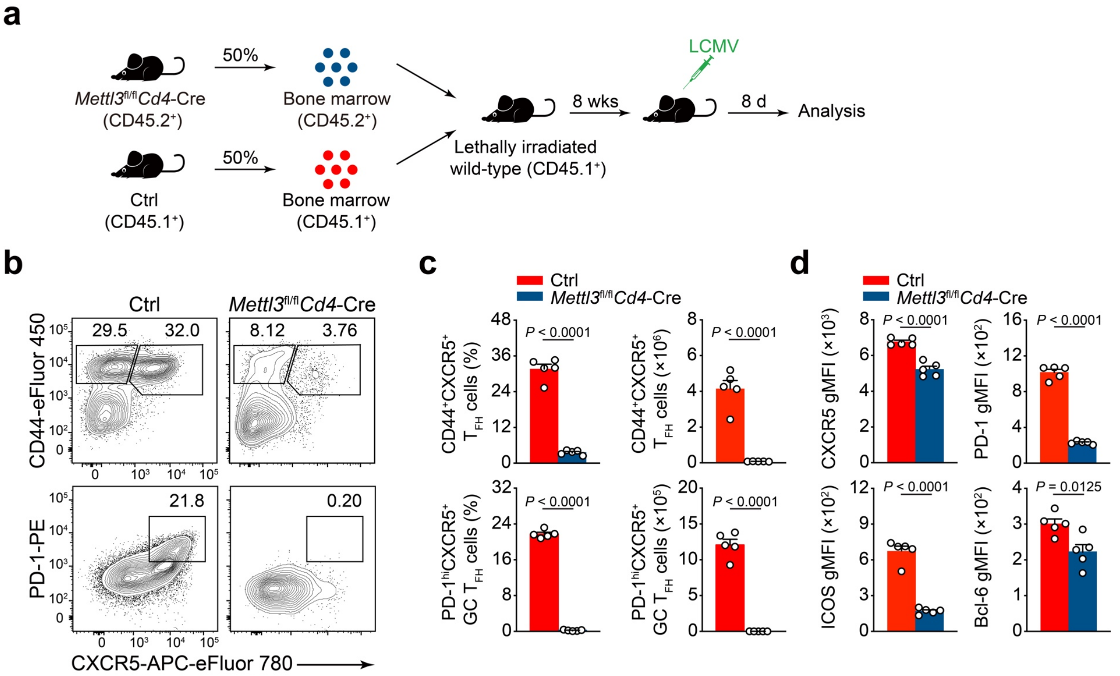

Supplementary Figure 3 METTL3 intrinsically regulates T$_{FH}$ differentiation.

a Generation of bone marrow (BM) chimera. 5×10^6 cells of a 1:1 mixture of *Mettl3*$^{fl/fl}$*Cd4*-Cre (CD45.2$^+$) and Ctrl (CD45.1$^+$) competitor BM cells were transferred into lethally irradiated wild-type (CD45.1$^+$) recipient mice. After 8 weeks reconstitution, recipient mice were infected with LCMV-Armstrong and analyzed by flow cytometry.

b, c Flow cytometry analysis of CD44$^+$CXCR5$^+$ T$_{FH}$ cells gated on CD4$^+$ T cells (top panel) and PD-1^{hi}CXCR5$^+$ GC T$_{FH}$ cells gated on CD44^{hi}CD62L^{lo}CD4$^+$ T cells (bottom panel) from the mixed BM chimeric mice on 8 *dpi*. Summary of the frequency and cell numbers of T$_{FH}$ cells and GC T$_{FH}$ cells are shown in **c** (n = 5 per group).

d Quantification of gMFIs of CXCR5, PD-1, ICOS, and Bcl-6 on donor CD44$^+$CXCR5$^+$ T$_{FH}$ cells (n = 5 per group).

Data are representative of two independent experiments. Error bars indicate standard error of the mean. P value was calculated by unpaired two-tailed Student's t-test.

Yao et al. Supplementary Figure 4

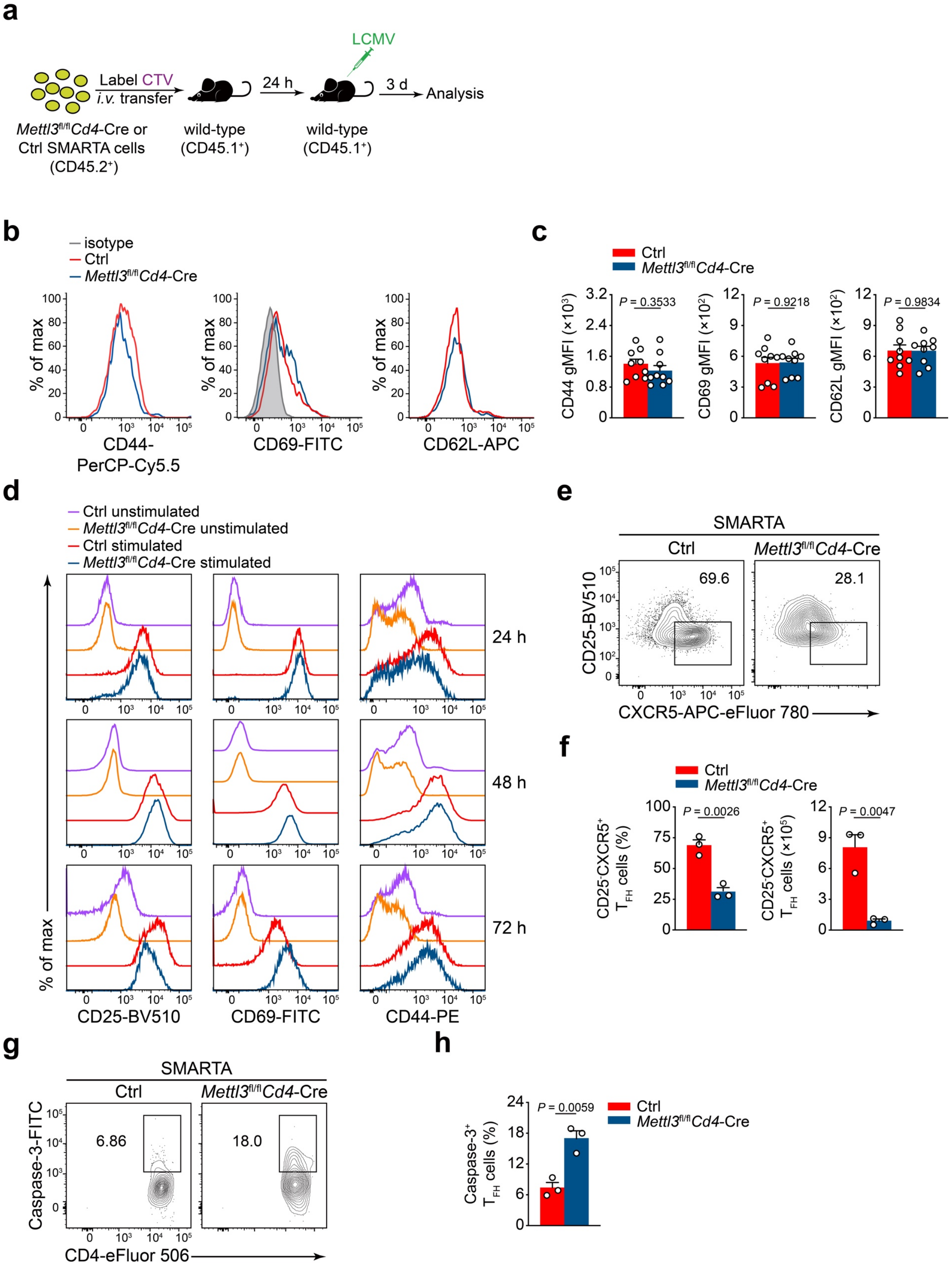

Supplementary Figure 4 METTL3 is necessary for the early T_{FH} differentiation.

a Scheme of adoptive transfer model. Naïve CTV-labeled SMARTA cells were adoptively transferred into wild-type recipients (CD45.1^{+}), followed by LCMV-

Armstrong infection *i.v.*, and splenic cells were analyzed on day 3 post infection by flow cytometry.

b Flow cytometry analysis of expression levels of CD44, CD69, and CD62L on SMARTA $CD4^+$ T cells from recipient mice as in **a**.

c Quantification of the gMFIs of CD44, CD69, and CD62L on SMARTA $CD4^+$ T cells as described in **b**, $n = 9$ per group.

d Splenic $CD4^+$ T cells from *Mettl3*$^{fl/fl}$*Cd4*-Cre or Ctrl mice were left unstimulated or stimulated with α-CD3/CD28 for 3 days. Histograms showing CD25, CD69, and CD44 expression on SMARTA $CD4^+$ T cells, $n = 3$ per group.

e, f Flow cytometry analysis of $CXCR5^+CD25^-$ T_{FH} cells gated on SMARTA $CD4^+$ T cells from recipient mice as in **a**. Summary of the frequency and cell numbers of T_{FH} cells are shown in **f**, $n = 3$ per group.

g, h Flow cytometry analysis of $Caspase\text{-}3^+CXCR5^+CD25^-$ T_{FH} cells among SMARTA cells from recipient mice as in **a**. Summary of the frequency and cell numbers of T_{FH} cells are shown in **h**, $n = 3$ per group.

Data are representative of three independent experiments (**d-h**) or pooled from three independent experiments (**b, c**). Error bars indicate standard error of the mean. *P* value was calculated by unpaired two-tailed Student's *t*-test.

Yao et al. Supplementary Figure 5

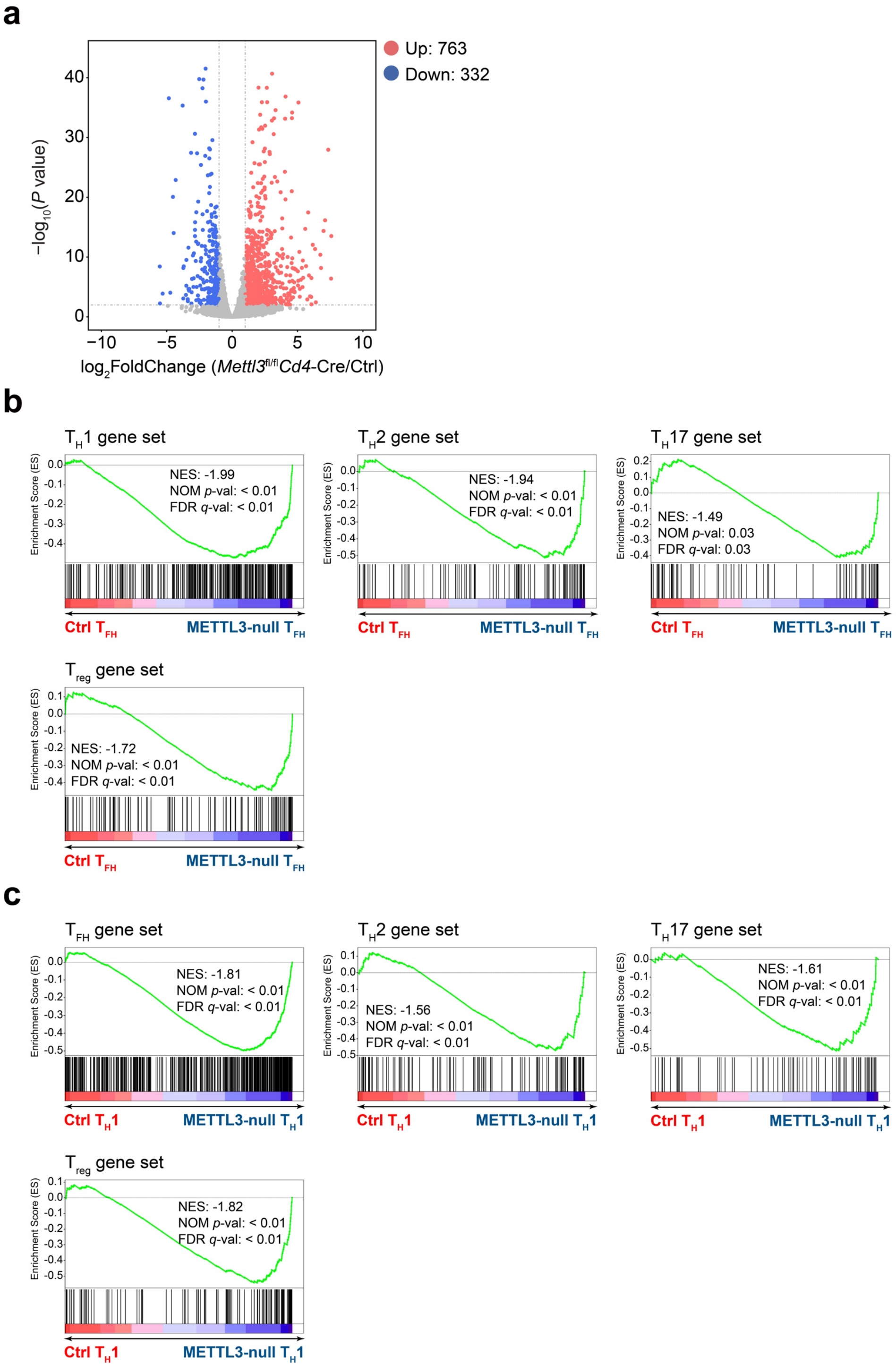

Supplementary Figure 5 GSEA analysis.

a Volcano map depicting genes upregulated (red) or downregulated (blue) 2-fold or more in T_H1 cells on 8 *dpi*. *P* value was calculated by Wald test.

b GSEA enrichment plots showing T_H1, T_H2, T_H17, and Treg-associated gene sets are enriched in *Mettl3*$^{fl/fl}$*Cd4*-Cre T_{FH} cells. *P* value was calculated by multiple hypothesis testing.

c GSEA enrichment plots showing T_{FH}, T_H2, T_H17, and Treg-associated gene sets are enriched in *Mettl3*$^{fl/fl}$*Cd4*-Cre T_H1 cells. *P* value was calculated by multiple hypothesis testing.

Yao et al. Supplementary Figure 6

a

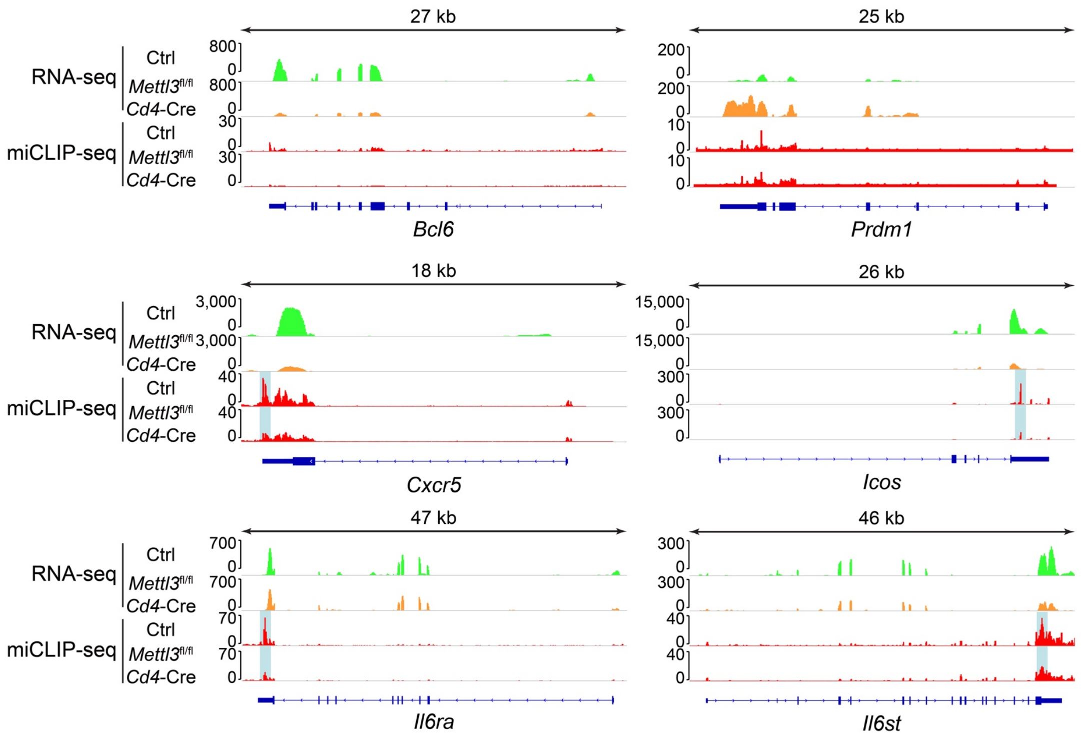

b

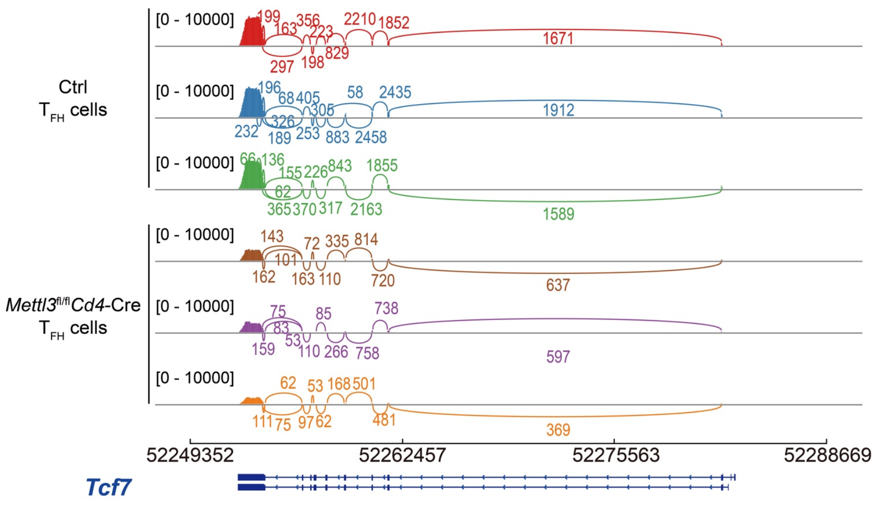

Supplementary Figure 6 m^{6}A directly modifies a series of T$_{FH}$ cell-associated transcripts.

a IGV tracks displaying RNA-seq (top panel) and m^{6}A-miCLIP-SMARTer-seq (bottom panel) reads distribution of interested genes. The baby blue highlight denotes significant m^{6}A peak.

b Sashimi plot showing read coverage and the reads that map to exon-exon junctions with arches. The numbers on the arches are the number of reads that map to that exon-exon junction.

Yao et al. Supplementary Figure 7

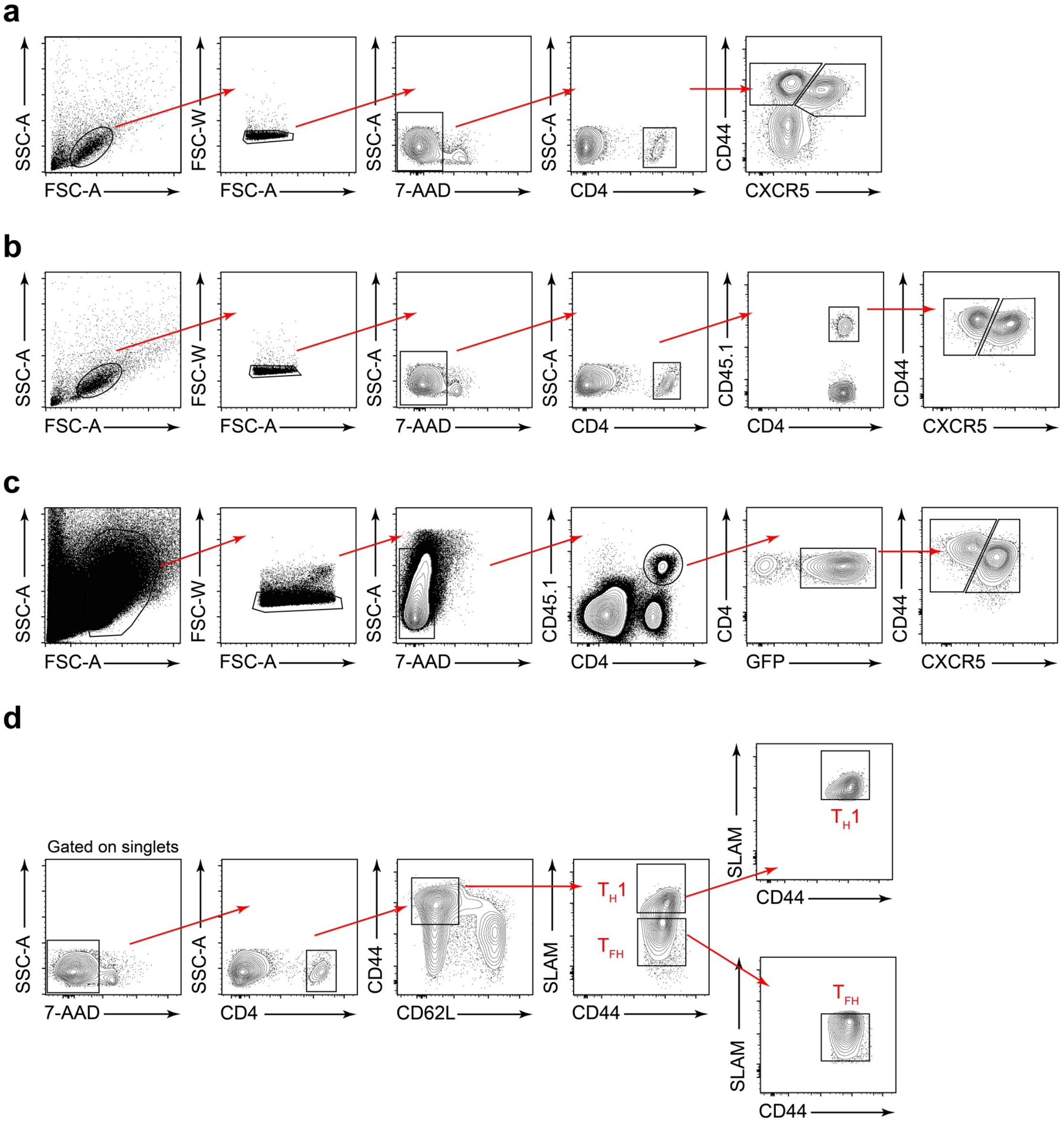

Supplementary Figure 7 Gating strategy for flow cytometry analysis and cell sorting.

a Analysis of polyclonal T_{FH} cell responses to LCMV-Armstrong infection and immunization (related to Figure 1, 2, S1, S2, and S3).

b Analysis of monoclonal T_{FH} cell responses to LCMV-Armstrong infection (related to Figure 2, 3, and S4).

c Analysis of T_{FH} cell responses after retroviral transduction (related to Figure 5 and 7).

d Because METTL3 deficiency affects CXCR5 expression, for RNA-seq analysis, $CD44^+SLAM^{hi}$ T_H1 cells and $CD44^+SLAM^{lo}$ T_{FH} cells were sorted for further analysis (related to Figure 4 and S5).

Yao et al. Supplementary Figure 8

a

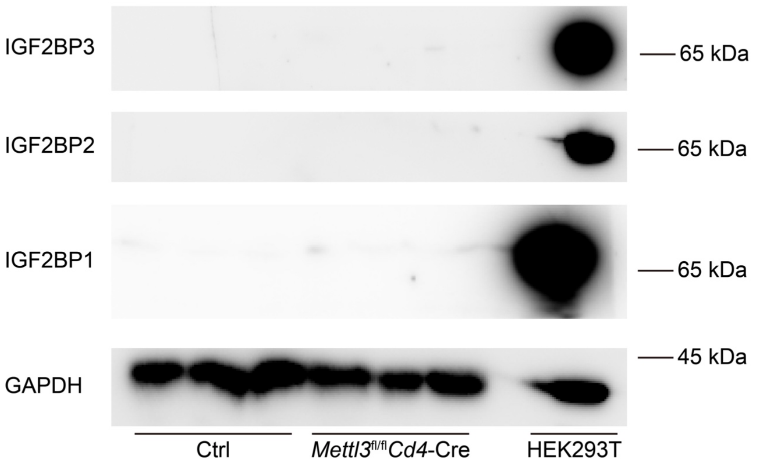

b

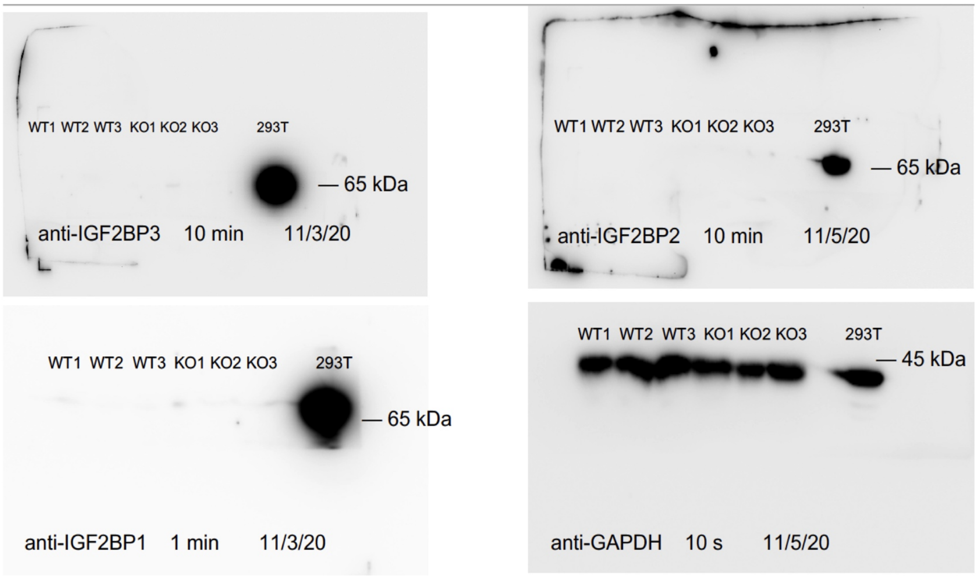

Supplementary Figure 8 Analysis of IGF2BPs by immunoblotting.

a CD4^{+} T cells sorted from Ctrl or *Mettl3*$^{fl/fl}$*Cd4*-Cre mice together with HEK293 T cells were subjected to Western blotting with antibodies specific for IGF2BP1, IGF2BP2, IGF2BP3, and GAPDH. Data are representative of two independent experiments.

b Uncropped images for **a**.

Yao et al. Supplementary Table 1

Primers for quantitative RT-PCR, m^{6}A-RIP-qPCR, and RIP-qPCR.

Primers for quantitative RT-PCR		
Gene	5' primer (5'→3')	3' primer (5'→3')
Gapdh	ACTCCACTCACGGCAAATTCA	GGCCTCACCCCATTTGATG
Hprt1	GCGTCGTGATTAGCGATGATG	CTCGAGCAAGTCTTTCAGTCC
Bcl6	AGACGCACAGTGACAAACCA	AGTGTGGGTCTTCAGGTTGG
Tcf7	CCCTTCCTGCGGATATAGAC	GGTACACCAGATCCCAGCAT
Lef1	TGAGTGCACGCTAAAGGAGA	CTGACCAGCCTGGATAAAGC
Cxcr5	CATGGGCTCCATCACATACA	GGCATGAATACCGCCTTAAA
Pdcd1	CTGGTCATTCACTTGGGCTG	AAACCATTACAGAAGGCGGC
Icos	TGCCGTGTCTTTGTCTTCTG	CTTCCCTTGGTCTTGGTGAG
Gzmb	CAAAGACCAAACGTGCTTCC	CTCAGCTCTAGGGACGATGG
Prdm1	ACAGAGGCCGAGTTTGAAGAGA	AAGGATGCCTCGGCTTGAA
Batf	CACAGAAAGCCGACACCCTT	CTCGGTGAGCTGTTTGATCTCT
Slamf6	CCCTGGAATGCAGTATGGTT	GCTCTGGGAGGACTCTGGAT
Socs1	CCGCTCCCACTCCGATTA	GCACCAAGAAGGTGCCCA
Mettl3	TAGTTCTAGGCTGGGGAGGT	ATCACTACGGAAGGTTGGGG
Id2	GTCCTTGCAGGCATCTGAAT	TTCAACGTGTTCTCCTGGTG
Il6ra	GCAAGAATCCTCGTCCATGT	GTGGAGGAGAGGTCGTCTTG
Bax	GAACCATCATGGGCTGGACAC	TGGTCACTGTCTGCCATGTG
Bcl2l11	CGACAGTCTCAGGAGGAACC	CATTTGCAAACACCCTCCTT
Primers for m^{6}A-RIP-qPCR and RIP-qPCR		
m^{6}A-Tcf7	TCCGATGACAGTGCTCTAGG	AAGAGTGAGAGCTGCAGAG
