# Supplementary material for: METTL3-dependent m6A modification programs T follicular helper cell differentiation
Source: Nat Commun. 2021 Feb 26;12:1333. doi: 10.1038/s41467-021-21594-6 (PMC7910450; doi:10.1038/s41467-021-21594-6)
Supplement: Supplementary file 3 — Reporting Summary [file 41467_2021_21594_MOESM3_ESM.pdf]

nature research

Corresponding author(s): Shuyang Yu

Last updated by author(s): Jan 19, 2021

Reporting Summary

Nature Research wishes to improve the reproducibility of the work that we publish. This form provides structure for consistency and transparency in reporting. For further information on Nature Research policies, see our Editorial Policies and the Editorial Policy Checklist.

Statistics

For all statistical analyses, confirm that the following items are present in the figure legend, table legend, main text, or Methods section.

n/a	Confirmed	
[ ]	[x]	The exact sample size (*n*) for each experimental group/condition, given as a discrete number and unit of measurement
[ ]	[x]	A statement on whether measurements were taken from distinct samples or whether the same sample was measured repeatedly
[ ]	[x]	The statistical test(s) used AND whether they are one- or two-sided<br>*Only common tests should be described solely by name; describe more complex techniques in the Methods section.*
[x]	[ ]	A description of all covariates tested
[x]	[ ]	A description of any assumptions or corrections, such as tests of normality and adjustment for multiple comparisons
[ ]	[x]	A full description of the statistical parameters including central tendency (e.g. means) or other basic estimates (e.g. regression coefficient) AND variation (e.g. standard deviation) or associated estimates of uncertainty (e.g. confidence intervals)
[ ]	[x]	For null hypothesis testing, the test statistic (e.g. *F*, *t*, *r*) with confidence intervals, effect sizes, degrees of freedom and *P* value noted<br>*Give P values as exact values whenever suitable.*
[x]	[ ]	For Bayesian analysis, information on the choice of priors and Markov chain Monte Carlo settings
[x]	[ ]	For hierarchical and complex designs, identification of the appropriate level for tests and full reporting of outcomes
[x]	[ ]	Estimates of effect sizes (e.g. Cohen's *d*, Pearson's *r*), indicating how they were calculated

Our web collection on statistics for biologists contains articles on many of the points above.

Software and code

Policy information about availability of computer code

Data collection	BD FACSVerse with FACSSuite software (v1.0.5) , BD LSRFortessa with FACSDiva software (v8.0.2), Bio-Rad CFX96 Connect Real-Time System, GloMax® 20/20 Luminometer
Data analysis	FlowJo v10, Graphpad Prism v8.0, Microsoft Excel v16.45, Image J v1.52g, Imaris (v8.1), FastQC v0.11.5, TopHat v2.1.1, HTseq v0.6.1, DESeq2 v1.18.1, fastq2collapse v1.1.3, Cutadapt v1.17, BWA v0.7.17, GSEA v4.1.0

For manuscripts utilizing custom algorithms or software that are central to the research but not yet described in published literature, software must be made available to editors and reviewers. We strongly encourage code deposition in a community repository (e.g. GitHub). See the Nature Research guidelines for submitting code & software for further information.

Data

Policy information about availability of data

All manuscripts must include a data availability statement. This statement should provide the following information, where applicable:

- Accession codes, unique identifiers, or web links for publicly available datasets
- A list of figures that have associated raw data
- A description of any restrictions on data availability

RNA-seq and m6A-miCLIP-SMARTer-seq datasets have been deposited in Gene Expression Omnibus (GEO) under the accession number GSE129650 (https://www.ncbi.nlm.nih.gov/geo/query/acc.cgi?acc=GSE129650). All data are available from the corresponding author upon reasonable request. Source data are provided with this paper.

Field-specific reporting

Please select the one below that is the best fit for your research. If you are not sure, read the appropriate sections before making your selection.

- [x] Life sciences
- [ ] Behavioural & social sciences
- [ ] Ecological, evolutionary & environmental sciences

For a reference copy of the document with all sections, see nature.com/documents/nr-reporting-summary-flat.pdf

Life sciences study design

All studies must disclose on these points even when the disclosure is negative.

Sample size	Sample size was not predetermined but we performed experiments with group sizes based on existing published literature of similar experiments. For animal experiments, n ≥ 3 was chosen based on the previous publications in the field (Li et al., Nat Commun, 2018 ; Xu et al., Nat Immunol, 2015 ; Xu et al., Immunity, 2017). For experiments other than those involving animals, n ≥ 3 was chosen based on the previous publications in the field (Wang et al., Nat Commun, 2019 ; Paris et al., Cell Stem Cell, 2019) and also because this size is necessary to calculate statistical significances.
Data exclusions	No data point excluded.
Replication	Every experiment was repeated at least 2 times, and all experiments were replicated successfully.
Randomization	For animal studies, age and sex matched animals were assigned randomly to each experimental and control group where applicable.
Blinding	Investigators were not blinded to group allocation during data collection. And there was no blinding in analysis of the experimental data based on experiment types (without subjective estimation, e.g. flow cytometry).

Reporting for specific materials, systems and methods

We require information from authors about some types of materials, experimental systems and methods used in many studies. Here, indicate whether each material, system or method listed is relevant to your study. If you are not sure if a list item applies to your research, read the appropriate section before selecting a response.

Materials & experimental systems

n/a	Involved in the study	
[ ]	[x]	Antibodies
[ ]	[x]	Eukaryotic cell lines
[x]	[ ]	Palaeontology and archaeology
[ ]	[x]	Animals and other organisms
[x]	[ ]	Human research participants
[x]	[ ]	Clinical data
[x]	[ ]	Dual use research of concern

Methods

n/a	Involved in the study	
[x]	[ ]	ChIP-seq
[ ]	[x]	Flow cytometry
[x]	[ ]	MRI-based neuroimaging

Antibodies

Antibodies used

The following fluorescence-labeled monoclonal antibodies were used:
anti-CD19 (PE-Cy7, clone 1D3, Thermo Fisher Scientific),
anti-CD25 (PE, clone PC61.5, Thermo Fisher Scientific),
anti-CD4 (PE-Cy7 or APC-eFluor780 or eFluor506, clone RM4-5, Thermo Fisher Scientific),
anti-CD44 (FITC or APC, clone IM7, Thermo Fisher Scientific),
anti-CD45.1 (PerCp-Cy5.5, clone A20, Thermo Fisher Scientific),
anti-CD45.2 (APC, clone 104, Thermo Fisher Scientific),
anti-CD62L (APC, clone MEL-14, Thermo Fisher Scientific),
anti-CD69 (FITC, clone H1.2F3, Thermo Fisher Scientific),
anti-CD8a (PE-Cy7 or biotin, clone 53-6.7, Thermo Fisher Scientific),
anti-B220 (FITC or PerCP-Cy5.5 or biotin, clone RA3-6B2, Thermo Fisher Scientific),
anti-GITR (APC, clone DTA-1, Thermo Fisher Scientific),
anti-GL7 (eFluor660, clone GL7, Thermo Fisher Scientific),
anti-PD-1 (PE, clone J43, Thermo Fisher Scientific),
anti-TCR Va2 (PE or APC, clone B20.1, Thermo Fisher Scientific),
anti-Gr.1 (biotin, clone RB6-8C5, Thermo Fisher Scientific),
anti-CD11b (biotin, clone M1/70, Thermo Fisher Scientific),
anti-CD11c (biotin, clone N418, Thermo Fisher Scientific),

anti-TER119 (biotin, clone TER-119, Thermo Fisher Scientific),
anti-CD49b (biotin, clone DX5, Thermo Fisher Scientific),
anti-Foxp3 (PerCp-Cy5.5 or APC, clone FJK-16S, Thermo Fisher Scientific),
anti-GATA3 (PE-Cy7, clone TWAJ, Thermo Fisher Scientific),
anti-RORγt (PE, clone AFKJS-9, Thermo Fisher Scientific),
anti-IL-4 (PE-Cy7, clone 11B11, Thermo Fisher Scientific),
anti-IgD (APC, clone 11-26c, Thermo Fisher Scientific),
anti-Bcl6 (PE, clone K112-91,BD Biosciences),
anti-CD138 (PE or BV421, clone 281-2, BD Biosciences),
anti-Fas (PE, clone Jo2, BD Biosciences),
and IL-17a (PE, clone TC11-18H10, BD Biosciences),
anti-SLAM (PE or APC, clone TC15-12F12.2, BioLegend),
anti-ICOS (PE-Cy7, clone C398.4A, BioLegend),

The following antibodies were used for CXCR5 staining:
purified anti-CXCR5 (clone G8, BD Biosciences),
biotin-conjugated goat anti-rat IgG (Cat# 112-066-143, Jackson Immunoresearch).

The following antibodies were used for intracellular staining:
TCF-1 Rabbit mAb (C63D9, cell Signaling Technology),
METTL3 Rabbit mAb (ab195352, abcam).

The following antibody was used for ELISA:
HPR-conjugated goat-anti rat IgG antibodies (A90-131P-39, Bethyl laboratories)

The following antibody was used for m6A-miCLIP-SMARTer-seq and m6A-RIP:
anti-m6A (ab151230, Abcam).

Validation

Antibody reagents are commercially available. Antibodies used in specific species have been appropriately validated by manufacturers and is information is provided on their website and data sheets as follows:
anti-CD19 (PE-Cy7, clone 1D3, Thermo Fisher Scientific), PMID: 26634606, PMID: 27064283;
anti-CD25 (PE, clone PC61.5, Thermo Fisher Scientific), PMID: 27551157, PMID: 28059703;
anti-CD4 (PE-Cy7 or APC-eFluor780 or eFluor506, clone RM4-5, Thermo Fisher Scientific), PMID: 27425374, PMID: 27376471, PMID: 27049058, PMID: 25642823, PMID: 27376549, PMID: 21333553;
anti-CD44 (FITC or APC, clone IM7, Thermo Fisher Scientific), PMID: 29174331, PMID: 29042531, PMID: 29030115, PMID: 27139492;
anti-CD45.1 (PerCp-Cy5.5, clone A20, Thermo Fisher Scientific), PMID: 25981357, PMID: 25642823;
anti-CD45.2 (APC, clone 104, Thermo Fisher Scientific), PMID: 27811056, PMID: 27322054;
anti-CD62L (APC, clone MEL-14, Thermo Fisher Scientific), PMID: 27064903, PMID: 25407678;
anti-CD69 (FITC, clone H1.2F3, Thermo Fisher Scientific), PMID: 27376549, PMID: 26436647;
anti-CD8a (PE-Cy7 or biotin, clone 53-6.7, Thermo Fisher Scientific), PMID: 28194013, PMID: 27760048;
anti-B220 (FITC or PerCP-Cy5.5 or biotin, clone RA3-6B2, Thermo Fisher Scientific), PMID: 27551155, PMID: 26551682, PMID: 27222343, PMID: 26523376, PMID: 24963139, PMID: 24747745;
anti-GITR (APC, clone DTA-1, Thermo Fisher Scientific), PMID: 27116251, PMID: 26551682;
anti-GL7 (eFluor660, clone GL7, Thermo Fisher Scientific), PMID: 25875847, PMID: 29042531;
anti-PD-1 (PE, clone J43, Thermo Fisher Scientific), PMID: 25348003, PMID: 23516361;
anti-TCR Va2 (PE or APC, clone B20.1, Thermo Fisher Scientific), PMID: 20479114, PMID: 19805617, PMID: 20089700, PMID: 19907079;
anti-Gr.1 (biotin, clone RB6-8C5, Thermo Fisher Scientific), PMID: 26807527, PMID: 27839867;
anti-CD11b (biotin, clone M1/70, Thermo Fisher Scientific), PMID: 28355185, PMID: 28112734;
anti-CD11c (biotin, clone N418, Thermo Fisher Scientific), PMID: 27716507, PMID: 26974160;
anti-TER119 (biotin, clone TER-119, Thermo Fisher Scientific), PMID: 28479188, PMID: 28461481;
anti-CD49b (biotin, clone DX5, Thermo Fisher Scientific), PMID: 24284909, PMID: 23420878;
anti-Foxp3 (PerCp-Cy5.5 or APC, clone FJK-16S, Thermo Fisher Scientific), PMID: 27565342, PMID: 27498556, PMID: 26809474, PMID: 26416167;
anti-GATA3 (PE-Cy7, clone TWAJ, Thermo Fisher Scientific), PMID: 28747424, PMID: 28844693;
anti-RORγt (PE, clone AFKJS-9, Thermo Fisher Scientific), PMID: 28262351, PMID: 28276457;
anti-IL-4 (PE-Cy7, clone 11B11, Thermo Fisher Scientific), PMID: 26621452, PMID: 18714021;
anti-IgD (APC, clone 11-26c, Thermo Fisher Scientific), PMID: 25613378, PMID: 23023393;
anti-Bcl6 (PE, clone K112-91,BD Biosciences), PMID: 21636296, PMID: 19608860;
anti-CD138 (PE or BV421, clone 281-2, BD Biosciences), PMID: 11466358, PMID: 10684261, PMID: 2519615, PMID: 2494194;
anti-Fas (PE, clone Jo2, BD Biosciences), PMID: 9620682, PMID: 1372394;
and IL-17a (PE, clone TC11-18H10, BD Biosciences), PMID: 9834129, PMID: 8877732
anti-SLAM (PE or APC, clone TC15-12F12.2, BioLegend), PMID: 16940424, PMID: 17406558, PMID: 17360942, PMID: 17237411;
anti-ICOS (PE-Cy7, clone C398.4A, BioLegend), PMID: 21964024, PMID: 25995205;
purified anti-CXCR5 (clone G8, BD Biosciences), PMID: 26214741, PMID: 30575739;
biotin-conjugated goat anti-rat IgG (Cat# 112-066-143, Jackson Immunoresearch), PMID: 26214741, PMID: 28604718;
TCF-1 Rabbit mAb (C63D9, cell Signaling Technology), PMID: 30575739; PMID: 30420627;
METTL3 Rabbit mAb (ab195352, abcam), PMID: 30401835, PMID: 30297870;
HPR-conjugated goat-anti rat IgG antibodies (A90-131P-39, Bethyl laboratories), PMID: 25282160, PMID: 25267791;

anti-m6A (ab151230, Abcam), PMID: 28809392, PMID: 30297870.

Eukaryotic cell lines

Policy information about cell lines

Cell line source(s)	HEK293T cell lines were purchased from ATCC.
Authentication	HEK293T cell lines were authenticated by STR profiling (for human lines) and species identification for validation by manufacturer. The cell lines were not authenticated in this study except cell morphology by microscopy.
Mycoplasma contamination	HEK293T cell lines were negative for mycoplasma test in the study .
Commonly misidentified lines (See ICLAC register)	No commonly misidentified cell lines were used in this study.

Animals and other organisms

Policy information about studies involving animals; ARRIVE guidelines recommended for reporting animal research

Laboratory animals	6- to 10-week-old C57BL/6J genetic background mice (both male and female) were used in this study: SMARTA, Cd4-Cre, ERT2-Cre, Mettl3fl/flCd4-Cre mice and Mettl3fl/flERT2-Cre mice. C57BL/6J (CD45.1+ and CD45.2+) mice were also used as recipients. All mice were kept in group housing (3–5 mice per cage) in a specific pathogen-free facility with controlled environmental conditions of humidity (50 ± 10%) , lighting (a 12-h light/dark cycle) and controlled temperature (21 ± 1 °C) at China Agricultural University.
Wild animals	No wild animals were used in this study.
Field-collected samples	No filed-collected samples were used in this study.
Ethics oversight	All mice in this study were handled in accordance with the protocol of Institutional Animal Care and Use Committee of China Agricultural University.

Note that full information on the approval of the study protocol must also be provided in the manuscript.

Flow Cytometry

Plots

Confirm that:

- [x] The axis labels state the marker and fluorochrome used (e.g. CD4-FITC).
- [x] The axis scales are clearly visible. Include numbers along axes only for bottom left plot of group (a 'group' is an analysis of identical markers).
- [x] All plots are contour plots with outliers or pseudocolor plots.
- [x] A numerical value for number of cells or percentage (with statistics) is provided.

Methodology

Sample preparation	Spleens or lymph nodes were mechanically digested, filtered into single cell suspensions, and stained using antibodies.
Instrument	Data were collected on a FACSVerse (BD Biosciences) with FACSSuite software (v1.0.5) or an LSRFortessa (BD Biosciences) with FACSDiva software (v8.0.2). BD Aria II (BD Biosciences) with FACSDiva software (v7.0) were used for cell sorting.
Software	We used the software FACSSuite (v1.0.5) and FACSDiva (v8.0.2 or v7.0; BD Biosciences) to collect the data and the software FlowJo (v10; Treestar) to analyze the data.
Cell population abundance	Purity was > 90% as determined by flow-cytometry, using a fraction of the sorted samples or enriched cells.
Gating strategy	Tfh cells were gated on FSC-A/SSC-A, FSC-W/FSC-H, SSC-W/SSC-H, 7-AAD-CD4+CXCR5+CD44+cells.<br>Th1 cells were gated on FSC-A/SSC-A, FSC-W/FSC-H, SSC-W/SSC-H, 7-AAD-CD4+CXCR5+CD44-cells.<br>GC Tfh cells were gated on FSC-A/SSC-A, FSC-W/FSC-H, SSC-W/SSC-H, CD4+CD44+CD62L-CXCR5+Bcl6-hi (or PD-1-hi or ICOS-hi) cells.<br>Plasma cells were gated on FSC-A/SSC-A, FSC-W/FSC-H, SSC-W/SSC-H, 7-AAD-CD138+IgD-lo cells.<br>GC B cells were gated on FSC-A/SSC-A, FSC-W/FSC-H, SSC-W/SSC-H, 7-AAD-B220+CD19+Fas+GL7+ (or PNA+) cells.

- [x] Tick this box to confirm that a figure exemplifying the gating strategy is provided in the Supplementary Information.
